# Microbial Diversity and Connectivity in Deep-Sea Sediments of the South Atlantic Polar Front

**DOI:** 10.3389/fmicb.2019.00665

**Published:** 2019-04-09

**Authors:** Gilda Varliero, Christina Bienhold, Florian Schmid, Antje Boetius, Massimiliano Molari

**Affiliations:** ^1^Max Planck Institute for Marine Microbiology, Bremen, Germany; ^2^School of Biological Sciences, University of Bristol, Bristol, United Kingdom; ^3^HGF-MPG Joint Research Group on Deep Sea Ecology and Technology, Alfred Wegener Institute for Polar and Marine Research, Bremerhaven, Germany; ^4^Helmholtz Centre for Ocean Research Kiel, GEOMAR, Kiel, Germany; ^5^MARUM Center for Marine Environmental Sciences, University of Bremen, Bremen, Germany

**Keywords:** Southwest Indian Ridge, seamounts, deep-sea, connectivity, diversity, bacteria, archaea

## Abstract

Ultraslow spreading ridges account for one-third of the global mid-ocean ridges. Their impact on the diversity and connectivity of benthic deep-sea microbial assemblages is poorly understood, especially for hydrothermally inactive, magma-starved ridges. We investigated bacterial and archaeal diversity in sediments collected from an amagmatic segment (10°–17°E) of the Southwest Indian Ridge (SWIR) and in the adjacent northern and southern abyssal zones of similar water depths within one biogeochemical province of the Indian Ocean. Microbial diversity was determined by 16S ribosomal RNA (rRNA) gene sequencing. Our results show significant differences in microbial communities between stations outside and inside the SWIR, which were mostly explained by environmental selection. Community similarity correlated significantly with differences in chlorophyll *a* content and with the presence of upward porewater fluxes carrying reduced compounds (e.g., ammonia and sulfide), suggesting that trophic resource availability is a main driver for changes in microbial community composition. At the stations in the SWIR axial valley (3,655–4,448 m water depth), microbial communities were enriched in bacterial and archaeal taxa common in organic matter-rich subsurface sediments (e.g., SEEP-SRB1, Dehalococcoida, Atribacteria, and Woesearchaeota) and chemosynthetic environments (mainly Helicobacteraceae). The abyssal stations outside the SWIR communities (3,760–4,869 m water depth) were dominated by OM1 clade, JTB255, Planctomycetaceae, and Rhodospirillaceae. We conclude that ultraslow spreading ridges create a unique environmental setting in sedimented segments without distinct hydrothermal activity, and play an important role in shaping microbial communities and promoting diversity, but also in connectivity among deep-sea habitats.

## Introduction

The deep seafloor beyond the shelf break comprises about 67% of the Earth’s lithosphere (Jørgensen and Boetius, 2007), making it the largest ecological realm worldwide. The increasing anthropogenic impact such as climate change, littering and industrial exploitation of deep-sea resources raises global concerns about the future of deep-sea biodiversity and the lack of marine conservation approaches for seabed habitats (Danovaro et al., 2017). In deep sea sediments, the largest fraction of taxonomic richness and biomass is contributed by members of the Bacteria and Archaea, which represent around 90% of the total benthic biomass and have a key role in organic matter remineralization and nutrient cycles (Jørgensen and Boetius, 2007; Wei et al., 2010). Thus, the investigation of spatial patterns of microbial diversity is crucial to better understand the mechanisms controlling the diversity and connectivity between deep-sea habitats. Deciphering factors that influence spatial turnover is relevant to the assessment of ecological function dynamics in the deep-sea. Advances in high-throughput 16S rRNA gene sequencing techniques have enabled the comparative analysis of microbial biogeographic patterns across marine environments (e.g., Sogin et al., 2006; Zinger et al., 2014), including the deep seafloor (Schauer et al., 2010; Durbin and Teske, 2011; Sylvan et al., 2012; Jacob et al., 2013; Anderson et al., 2015; Ristova et al., 2015; Ruff et al., 2015; Bienhold et al., 2016). Distance-decay relationships (i.e., a decrease in taxonomic similarity with increasing geographic distance) and a relatively high degree of endemism, investigated at various taxonomic resolution, have been reported both at local and regional (tens to hundreds of kilometers; Schauer et al., 2010; Jacob et al., 2013; Ishibashi et al., 2015; Shulse et al., 2016; Walsh et al., 2016) and at global scales (Ruff et al., 2015; Mino et al., 2017). Selection, drift, dispersal and mutation are the four evolutionary and ecological interplay processes that shape the microbial biogeography (Hanson et al., 2012). The presence of substantial endemism and distance-decay relationships has been interpreted as a rather rapid diversification (selection and drift) and limited dispersal across ocean basins (Hanson et al., 2012; Bienhold et al., 2016). Whilst environmental selection has been shown to play an important role in shaping deep-sea benthic microbial communities (e.g., Bienhold et al., 2012, 2016), the physical mechanisms responsible for dispersal limitation (e.g., currents and seafloor geomorphology) are poorly understood (Zinger et al., 2014). Patterns of deep-sea bacterial biogeography observed at the global scale suggest that seafloor geomorphology (i.e., mid-ocean ridges and oceanic trenches), deep-water masses and landmasses may represent barriers to dispersal (Nunoura et al., 2015; Bienhold et al., 2016; Salazar et al., 2016; Wenzhöfer et al., 2016).

Mid-ocean ridges (MOR) are undersea mountain ranges forming the largest continuous topographic feature on Earth, a global network almost 85,000 km long (Kennett, 1982). At active MOR oceanic lithosphere formation coincides with substantial fluxes of heat. Seafloor hydrothermal circulation is generated by downward percolation of seawater, through the fractured ocean crust, that is heated at depth. When the fluid becomes buoyant it rises rapidly back to the seafloor where it is expelled into the overlying water column. This fluid generates strong geochemical and physical gradients and provides a chemical energy source for microbial growth, which supports chemosynthesis-based food chains and promotes high physiological diversity (German et al., 2011; Sievert and Vetriani, 2012; Gollner et al., 2015; Goffredi et al., 2017). Despite the substantial passive dispersal of microbes in the ocean (De Rezende et al., 2013), there is increasing evidence that geochemistry and geographical isolation play a role in structuring vent microbial communities (e.g., Flores et al., 2012; Akerman et al., 2013; Campbell et al., 2013; Mino et al., 2017), as has been observed for vent macrofauna (e.g., Rogers et al., 2012). MOR vent fields have been estimated to occur every 25–90 km, but only a small fraction thereof have been mapped and investigated, indicating that microbial diversity and biogeography patterns are largely unknown for most of the ridge segments (Beaulieu et al., 2015). Even less it is known about the microbial diversity at inactive segments where surface expressions of hydrothermalism are absent. It has been proposed that ridges can be stepping stones and pathways for the dispersal of slope fauna into the open ocean and/or may act as barriers to the dispersal of abyssal seafloor fauna (Wilson and Kaufmann, 1987; Vinogradova, 1997; Dinter, 2001; Mironov, 2006; Gebruk et al., 2010), but no studies have yet investigated the role that mid-ocean ridges may play for the connectivity of deep-sea benthic microbial communities.

The Southwest Indian Ridge (SWIR) is a major plate boundary of the world oceans, separating the African and Antarctic plates and extending from the Bouvet triple junction (BTJ) in the South Atlantic Ocean to the east Rodrigues triple junction (RTJ) in the Indian Ocean (Sauter and Cannat, 2013). The SWIR segment in the southern Atlantic Ocean separates the Agulhas basin to the north and the Weddell/Enderby plains to the south, which belong to the same biogeochemical deep-sea floor province as defined by sedimentary organic carbon content, bottom hydrography (i.e., temperature and salinity) and organic matter flux (Seiter et al., 2004; Watling et al., 2013). Due to the presence of Antarctic cold dense bottom water masses flowing eastward, SWIR forms a barrier to the northward and southward flow of bottom water (Larqué et al., 1997; Haine et al., 1998; Orsi et al., 1999; Rutgers van der Loeff et al., 2016). Furthermore, the SWIR is an ultraslow-spreading ridge, which is characterized by low magma input and scant hydrothermal circulation, and by a deep ridge valley that is on average 4,000 m deep, with ridge flanks that rise up to 1,000 m depth (Dick et al., 2003). These features make the SWIR an interesting place to test whether ridges can limit the dispersal of deep-sea benthic microbial communities in the absence of hydrothermalism. Specifically, we assessed bacterial and archaeal community structure and diversity based on the 16S rRNA gene to investigate whether the western section of the SWIR (i) acts as a physical barrier between communities to the North and South, limiting microbial dispersal, and (ii) promotes isolation of microbial communities inside the ridge.

## Materials and Methods

### Sample Collection

**FIGURE 1:**
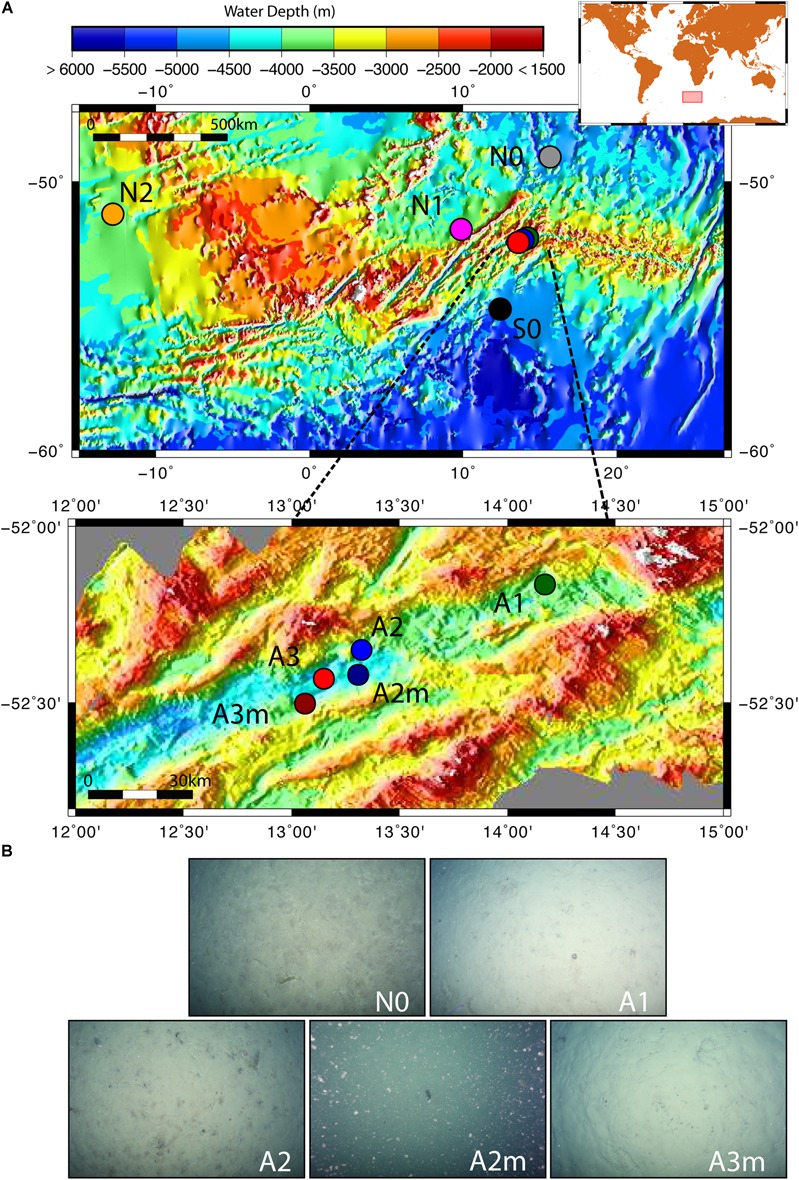
Map of the investigated sites and seafloor pictures. **(A)** The zoom-in shows the stations inside the Southwest Indian Ridge valley; **(B)** seafloor photos for station to the north of the SWIR (N0) and for stations inside the SWIR showing diatom ooze sediments; seafloor picture at A2m also shows resuspended sediment particles, which were likely responsible of turbidity signals measured at south ridge flank (Schlindwein, 2014). Green, blue and red dots represent stations inside SWIR, and orange, magenta, gray and black dots represent stations outside SWIR.

**FIGURE 2:**
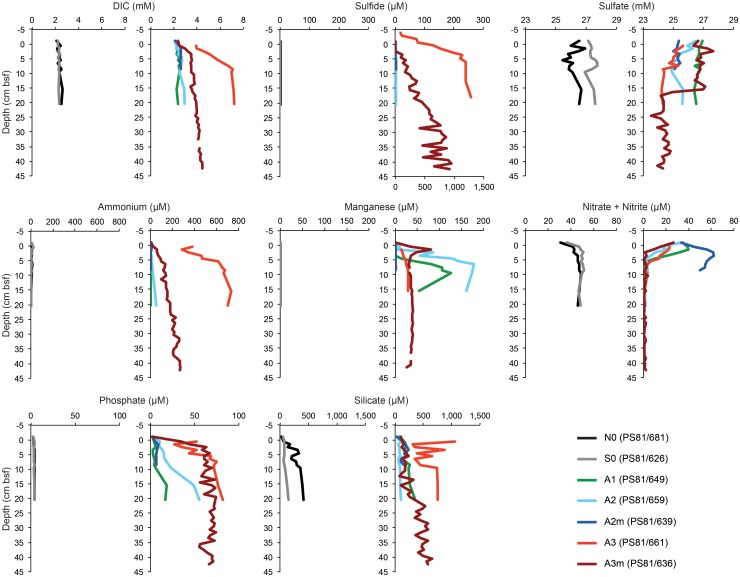
The porewater profiles of surface sediments (0–5 cm) outside and inside the SWIR. The gray (southern reference) and black lines (northern reference) represent outside SWIR stations, the colored lines inside samples. The sulfide plot values for station A3 (PS81/661) refer to the bottom *x*-axis, for all other stations the values refer to the top *x*-axis. DIC, dissolved inorganic carbon; cm bsf, centimeters below seafloor.

The sediment samples were collected in the segment 10°–17°E of the SWIR during the expedition ANTXXIX/8 with the research vessel Polarstern (PS81) in 2013 (Figure 1A). The SWIR segment studied here is an amagmatic accretionary ridge segment with a spreading rate of less than 15 mm yr^-1^ and the major percentage of the axial seafloor is constituted by mantle rocks (Dick et al., 2003). In the earlier investigation of this SWIR segment the presence of a hydrothermal plume has been suggested based on turbidity maxima in the water column (Bach et al., 2002). During the PS81 expedition we investigated the turbidity plumes but found them not linked to hydrothermal emissions (Schlindwein, 2014). Clear signals of white or black smoker type venting were lacking, suggesting that this system is a quiescent ridge segment. However, the ridge flanks and trough were heavily sedimented, indicating a productive surface ocean and a relatively substantial input of plankton debris, foremost diatom ooze (Figure 1B). Sediment samples have been taken from the seabed at a depth range of 3,655 and 4,869 m. Surface sediment samples, 0–40 cm below the seafloor (bsf), were collected with a TV-guided multi-corer device (TV-MUC) and subsurface samples, from ca. 50 to 600 cm bsf, with a gravity core (GC). For surface sediments two replicate TV-MUC cores were collected from each site, one used for porewater and one for sediment sampling. For subsurface sediments one GC was collected at A1, A2, and A3, and sediment and porewater were collected from the same GC (Table 1). Cores were sliced on board (0–1, 1–5, and 5–10 cm for MUC cores, and every 40 cm for GC cores) and sediment samples for DNA analysis were stored at -80°C. Porewater was collected at intervals of 1 cm, starting from the water overlying the sediment to the bottom of the core in MUC cores, and every 40 cm in GC cores. Sediments were sampled at 2 reference stations (S0 and N0), located to the south and north of the SWIR, respectively, and at 5 stations inside the SWIR valley (A1, A2, A2m, A3, and A3m). The stations inside the ridge were selected based on environmental data and visual observations: Area 1 (A1) had highest heat flow values (up to 1,000 mW m^-2^; Schlindwein, 2014); at Area 2 a signature for a hydrothermal plume had previously been reported by Bach et al. (2002), but only based on turbidity maxima in the water column, with stronger turbidity plumes at station A2m than at station A2 (Figure 1B); Area 3 represents the axial valley of the ridge, with station A3 sampled in the central part and station A3m located close to the site where a vesicomyid clam, a typical inhabitant of reduced chemosynthetic habitats, was discovered (Supplementary Figure S6). Additionally, in order to better investigate the effect of the geographical distance and the SWIR on benthic microbial diversity and connectivity in the South Atlantic Polar Front, the microbial communities were also investigated in sediments from two stations (N1 and N2) sampled during the Polarstern cruise PS79 in 2012 (Ruff et al., 2014), and located northwest of the SWIR segment investigated here (Figure 1). Major changes along porewater profiles occurred in the first top 5 cm of sediments (Figure 2 and Supplementary Figure S1), hence microbial communities were described for this top sediment layers (0–5 cm bsf) and for two subsurface layers (110 and 410 cm), the latters as representative of subsurface microbial community.

**Table 1:** Description of investigated sites.

	Sample	Sampling					Sediment								
Station	ID	time	Latitude	Longitude	Depth	Sediment type	layer	Chl-*a*	TOC	DIC	Sulfide	Ammonium	Nitrate	Methane	Porosity
			(N)	(E)	(m)		(cm bsf)	(μg/ml)	(μgC/mg)	(mM)	(μM)	(μM)	(μM)	(μM)	(ϕ)
PS79/141	N2	19.02.12	-51.268	-12.618	4114	Siliceous ooze	0.0–5.0	na	na	na	na	na	na	na	na
PS79/081	N1	19.01.12	-52.011	10.011	3760	Siliceous ooze	0.0–5.0	na	na	na	na	na	na	na	na
PS81/681	N0	11.12.13	-48.731	15.679	4351	Siliceous ooze	0.0–5.0	0.14	5.8	2.3 (2.1–2.4)	0	7.7 (9–4)	43.6 (41–46)	na	0.92
PS81/626	S0	22.11.13	-54.959	12.479	4869	Siliciclastic clay	0.0–5.0	0.04	4.6	2.3 (2.2–2.3)	0	8.6 (24–7)	47.4 (44–49)	na	0.83
PS81/649	A1	01.12.13	-52.168	14.177	3655	Siliceous ooze	0.0–5.0	0.24	4.0	2.3 (2.3–2.5)	0	13.1 (10–17)	27.2 (38–9)		0.98
PS81/659	A2	05.12.13	-52.368	13.320	3941	Siliceous ooze	0.0–5.0	0.30	4.1	2.4 (2.2–2.7)	0	4.0 (2–5)	10.5 (20–0)	0.2 (0.2–0.2)	0.95
PS81/639	A2m	28.11.13	-52.434	13.305	4375	Siliceous ooze	0.0–5.0	0.28	3.1	2.5 (2.1–2.6)	0	5.0 (10–1)	55.8 (43–62)	na	0.95
PS81/661	A3	05.12.13	-52.441	13.137	4415	Siliceous ooze	0.0–5.0	0.49	3.6	4.6 (3.9–5.5)	317 (89–643)	388 (285–468)	18.3 (23–14)	na	0.97
PS81/636	A3m	27.11.13	-52.497	13.065	4199	Siliceous ooze	0.0–5.0	0.33	4.9	3.1 (2.4–3.5)	8.9 (0–26)	62.0 (29–92)	5.4 (14–1)	0.3 (0.7–0.2)	0.95
PS81/653	A1	02.12.13	-52.17	14.181	3709	Siliceous ooze	110	0.35	3.0	2.0	5	31	1.67	0.1	0.86
PS81/656	A2	04.12.13	-52.366	13.317	3968	Siliceous ooze	110	0.42	3.8	3.8	14	153	0.4	0.2	0.91
PS81/657	A3	04.12.13	-52.441	13.135	4448	Siliceous ooze	110	0.70	4.9	15.2	4046	1603	0.0	0.8	0.96
PS81/653	A1	02.12.13	-52.17	14.181	3709	Siliceous ooze	410	0.23	3.3	1.9	0	17	0.7	0.1	0.85
PS81/656	A2	04.12.13	-52.366	13.317	3968	Siliceous ooze	410	0.23	4.2	3.8	28	216	0.0	0.2	0.83
PS81/657	A3	04.12.13	-52.441	13.135	4448	Siliceous ooze	410	0.73	4.1	16.6	4242	1711	0.0	0.9	0.87

*cm bsf, centimeters below seafloor; Chl-a, chlorophyll a; TOC, total organic carbon; DIC, dissolved inorganic carbon; na, not available. For all sedimentary parameters, average values for surface sediments (0–5 cm) are provided; for porewater data, the range along the profiles (0–1 and 4–5 cm) is indicated between brackets. For detailed profiles see Figure 2 and Supplementary Figures S1, S2.*

### Porewater and Sediment Biogeochemistry

For surface sediments, two replicate cores were collected from each site, one used for porewater and one for sediment sampling. For subsurface sediments, sediment and porewater were collected from the same gravity core. The subsampling of cores was performed immediately after recovery in a temperature controlled lab at 2°C. Profiles of dissolved components like inorganic carbon (DIC), nutrients [NH_4_^+^, PO_4_^3-^, NO_2-_, NO_3_^-^ + NO_2_^-^, Si(OH)_4_], sulfate, sulfide and manganese (Mn) in the sediments were assessed by extracting porewater with Rhizons (SMS type MOM 19.21.21F, mean pore size 0.15 μm; Rhizosphere Research Products). For DIC 2 mL porewater were filled headspace-free into glass vials leaving no headspace and stored at 4°C. DIC was assessed via flow injection analysis (Grasshoff et al., 2007). Nutrients were measured with a continuous Flow Nutrient Analyzer “QuAAtro39” (Seal Analytical) according to Grasshoff et al. (2007). Sulfide samples were fixed in plastic vials pre-filled with 0.5 mL 2% ZnAc before being stored at 4°C, and analysis was performed according to procedures described by Cline (1969). Porewater samples for Mn analysis were fixed in plastic vials pre-filled with 0.2 mL 1 M HCl before being stored at 4°C. Mn concentrations were assessed by atomic absorption spectrometry. Solid phase sediment samples were collected by slicing the core in 0–1, 1–5, and 5–10 cm layers, and were preserved for analyses of porosity, chloroplastic pigment equivalents (CPEs), total organic carbon (TOC) and total organic nitrogen (TON). Samples were prepared and analyzed as described in Böer et al. (2009). At the investigated water depths (3,655–4,869 m) no photosynthesis occurs, thus the sedimentary amount of chlorophyll pigments is used as a measure of the amount and freshness of phytodetritus sinking from the productive photic water layers.

For CH_4_ gas analysis 5 mL of sediment was collected with cut-off 5 mL syringes and added to 10 mL 2.5% NaOH in glass crimp vials, mixed, stored upside down at 4°C and then analyzed by gas chromatography (Focus GC, Thermo Fisher Scientific) as described in Thang et al. (2012). The entire biogeochemical dataset has been deposited in the PANGAEA database^1^.

We used Fick’s law to estimate the vertical diffusive flux *J* of geochemical constituents in the sediment cores,

$$J=\theta D_{s}\frac{\delta C}{\delta z}$$

Here ∅ is porosity, D_s_ = $\frac{D_{0}}{\theta }$ is the sediment diffusion coefficient, calculated from the sediment deviated tortuosity 𝜃 = 1.1 for a porosity of 0.9 (Matyka et al., 2008) and *D*_0_ is the tracer diffusion coefficient in seawater. We used values of *D*_0_ = 4.64 × 10^-6^ [cm^2^ s^-1^] for sulfate, *D*_0_ = 9.17 × 10^-6^ for sulfide, *D*_0_ = 9.03 × 10^-6^ [cm^2^ s^-1^] for ammonium, *D*_0_ = 3.02 × 10^-6^ [cm^2^ s^-1^] for manganese, *D*_0_ = 9.03 × 10^-6^ [cm^2^ s^-1^] for nitrate. Coefficients are taken from Schulz (2000). δC is the difference in concentration [mmol L^-1^] and δz is the difference in depth [m].

### Microbial Abundance and Activity

For microbial cell count, the top 1 cm of sediment in MUC cores was fixed in 2% buffered formaldehyde/water and stored at 4°C until subsequent analysis. Microbial abundance was estimated by epifluorescence microscopy after staining with Acridine Orange following the procedure described by Hobbie et al. (1977) and modified by Böer et al. (2009). Catalyzed reporter deposition fluorescence *in situ* hybridization (CARD-FISH) was applied to enumerate the active fraction of bacterial and archaeal assemblages (Amann and Fuchs, 2008). Samples were stored and processed according to the procedure of Ishii et al. (2004) and for archaeal cell-wall permeabilization according to Molari and Manini (2012). Hybridization conditions were applied as previously described for EUB338I-III, targeting members of the Bacteria (Amann et al., 1990; Daims et al., 1999), ARCH915 targeting most members of the Archaea (Stahl and Amann, 1991), and NON338 as negative control (Amann et al., 1995). For checking the reliability of the FISH signal the CARD-FISH filter was counter-stained with DAPI, and up to 700 EUB-FISH-stained cells and 100 ARCH-FISH-stained cells were counted per sample. The relative abundances of Bacteria and Archaea were based on total AODC counts, as the latter gives more reliable counts than DAPI in sediment composed by small particle size (<62.5 μm) (e.g., Schippers et al., 2005). The CARD-FISH efficiency (i.e., sum of bacterial and archaeal relative abundances) does not reach 100% of AODC counts, as not all cells are captured by FISH potentially due to incomplete coverage of probes, low ribosome content and lack of proper cell-wall permeabilization (Amann and Fuchs, 2008).

Total microbial activity was estimated by uptake of ^14^C-labeled inorganic carbon. Dark CO_2_ fixation (DCF) rates were estimated following the procedures described by Molari et al. (2013) including some modifications. DCF rates were measured incubating 1 mL of sediment slurry (∼1:1 mixture of sediment and filtrated 0.22-μm bottom seawater) in triplicate with 12 μL ^14^C-labeled sodium bicarbonate (0.25 mCi mL^-1^, final activity 3 μCi mL^-1^) in the dark at *in situ* temperature (2–4°C). The incubations were terminated by the addition of 1 mL formaldehyde in seawater (final concentration 2%) after 12 h. Two controls per sample were killed with 1 mL formaldehyde in seawater (final concentration 2%) before addition of the tracer. Samples were stored at 4°C until further processing. At MPI laboratory, the samples were centrifuged at 12,000 rpm for 5 min, the supernatant was discarded, and remaining sediment pellets were washed three times with 1× PBS. The sediment pellets were resuspended with 1 mL 3 M HCl, transferred into a new 50 mL vial and mixed constantly by bubbling with pressurized air for 4 h. The samples where mixed with 8 mL of the Scintillation cocktail Ultima Gold^TM^ and centrifuged at 3,500 rpm for 30 min. The supernatants were transferred into a 20 mL scintillation vials and the pellets were resuspended in 8 mL Ultima Gold^TM^ and centrifuged a second time. The supernatants were combined and measured with a liquid scintillation counter up to 10 min. The DPM were converted in moles of inorganic carbon incorporated per unit of sediment volume and time using the formula described by Molari et al. (2013).

### DNA Extraction and Sequencing

DNA was extracted from 1 g of homogenized sediment from 0–1, 1–5, 110, and 410 cm sediment layers using the FastDNA^TM^ SPIN Kit for Soil (Q-BIOgene, Heidelberg, Germany) following the manual protocol. Then, an isopropanol precipitation was performed on the extracted DNA, and DNA samples were stored at -20°C. DNA extracts from 0–1 to 1–5 cm were pooled at equal volumes and DNA amount prior to sequencing.

Amplicon sequencing was done at the CeBiTec laboratory (Centrum für Biotechnologie, Universität Bielefeld) on an Illumina MiSeq machine. For the 16S rRNA gene amplicon library preparation we used the bacterial primers 341F (5′-CCTACGGGNGGCWGCAG-3′) and 785R (5′-GACTACHVGGGTATCTAATCC-3′) and the archaeal primers Arch349F (5′-GYGCASCAGKCGMGAAW-3′) and Arch915R (5′-GTGCTCCCCCGCCAATTCCT-3′) (Wang and Qian, 2009; Klindworth et al., 2013) which amplify the 16S rRNA gene hypervariable region V3–V4 in Bacteria (400–425 bp fragment length) and the V3–V5 region in Archaea (510 bp fragment length). The amplicon library was sequenced with the MiSeq v3 chemistry, in a 2 × 300 bp paired run with >50,000 reads per sample, following the standard instructions of the 16S Metagenomic Sequencing Library Preparation protocol (Illumina, Inc., San Diego, CA, United States).

The quality cleaning of the sequences was performed with several software tools. Primer clipping was performed with cutadapt (Martin, 2011). TRIMMOMATIC (Bolger et al., 2014) was used to remove the sequences of low quality (for Bacteria SLIDINGWINDOW:4:10 MINLEN:300; for Archaea SLIDINGWINDOW:6:13 MINLEN:450); this step was performed before the merging of reverse and forward reads for the bacterial dataset and after the merging for the archaeal dataset in order to enhance the number of retained reads for long archaeal 16S fragments. The merging of forward and reverse reads was performed with PEAR (Zhang et al., 2014). Clustering of sequences into OTUs (operational taxonomic units) was done using the SWARM algorithm, based on one nucleotide difference between amplicons (parameter settings: -b 3 -d 1 -f; Mahé et al., 2014). The taxonomic classification was based on the SILVA 128 database (Quast et al., 2013). During this step, sequences with less than 90% of similarity with SILVA sequences were removed.

The total number of sequences obtained in this study is reported in Supplementary Table S1. Sequences were deposited at the European Nucleotide Archive (ENA) under accession number PRJEB23821; the sequences were archived using the service of the German Federation for Biological Data (GFBio; Diepenbroek et al., 2014). Absolute singletons (SSO_abs_), i.e., OTUs consisting of sequences occurring only once in the full dataset (Gobet et al., 2013), accounted for 92–98% of all OTUs (20–56% of all sequences) for bacterial and archaeal datasets, respectively, and they were not included in diversity and community analyses.

### Data Analysis

Alpha-diversity was assessed as species richness, exponential of Shannon index and inverse of Simpson index, corresponding to Hill’s numbers of order *q* = 0 (*H*_0_), *q* = 1 (*H*_1_) and *q* = 2 (*H*_2_), respectively (Hill, 1973; Chao et al., 2014). Estimated richness (Chao1), shared and unique OTUs (i.e., OTUs that are only present at one station) were calculated by rarifying the sequences to the smallest dataset (25,167 sequences for the domain Bacteria and 1,190 sequences for Archaea) 100 times and taking the average values, to account for differences in sequencing depth between samples. Non-parametric Mantel tests based on the Spearman correlation coefficient with significance assessed based on 1000 Monte Carlo permutations were used to determine correlations between genetic, spatial, and environmental distance matrices (Legendre and Legendre, 1998). To determine the strength of the relationship between similarity in community composition, geographic distance, and environmental settings linear models were fitted. Differences in microbial community composition were visualized with non-metric multidimensional scaling plots, and analysis of similarity (ANOSIM; Clarke, 1993) was used to assess significant differences between groups of samples from outside and within the ridge, and from surface and subsurface sediments. Redundancy analysis (RDA) in combination with variation partitioning (VP) was conducted to test the effect of environment variables on variations in microbial community composition. The analysis of microbial community composition was carried out on dominant bacterial and archaeal taxa, here defined as those composed by OTUs that represent more than 0.1% of the total number of sequences in each sample. Principal component analysis (PCA) was performed at bacterial class and family levels to identify which taxa were responsible for differences between stations. Prior to the analyses: (i) the environmental variables were standardized (i.e., *z*-scored) and filtered by collinearity based on the variance inflation factor (VIF) of less than five, which retained chlorophyll *a*, TOC and DIC; specifically DIC, ammonia, sulfide, and porosity were highly correlated (Pearson *r* > 0.95, *p* < 0.001), thus we selected DIC as a proxy for the presence of upward porewater fluxes carrying reduced compounds (e.g., ammonia and sulfide); (ii) the OTU dataset was standardized using the Hellinger transformation (Legendre and Gallagher, 2001). All analyses were carried out in the R statistical environment (R Development Core Team, 2013) with the packages vegan (Oksanen et al., 2016), ggplot2 (Wickham, 2009), devtools (Wickham and Chang, 2015), factoextra (Kassambara, 2015), ade4 (Dray and Dufour, 2007), plyr (Wickham, 2011), reshape (Wickham, 2007), and usdm (Naimi et al., 2014), as well as with custom R scripts. The rarefaction curves and the diversity analyses were performed with the iNEXT package (Hsieh et al., 2016) using default parameters (i.e., endpoint = double of the sample size; knots = 40).

The phylogenetic trees were constructed with the RAxML software (Stamatakis, 2014) and the R environment (R Development Core Team, 2013) using the ape (Paradis et al., 2004), phyloseq (McMurdie and Holmes, 2015), and ggplot2 (Wickham, 2009) packages. The sequences for the tree backbone were retrieved from the SILVA SSU Ref database (v128) and it was built with the maximum likelihood method (1,000 bootstraps). Sequences obtained in this study with Illumina tag sequencing were added to the tree backbone using the parsimony method.

## Results

### Sediment Biogeochemistry

At all sampled stations, the seafloor consisted of diatom ooze, with exception of the southernmost station (S0), where the sediment was siliciclastic clay (Table 1). The porewater profiles of surface sediments showed different patterns between the stations inside and outside the SWIR (Figure 2). Specifically, anomalies in DIC, ammonia, phosphate, and sulfide concentrations were observed at the SWIR western stations (Table 1), with steepest gradients at A3–A3m (Figure 2 and Supplementary Figure S1A). A depletion of nitrate below 0.1 m depth was observed in all cores from inside the SWIR valley, but not at the reference sites outside the valley (N0 and S0). The shallow nitrate depletion inside the axial valley suggests a lower oxygen penetration compared to the reference sites outside the axial valley. The concentration and C:N ratio of organic matter in surface sediments did not show remarkable differences between stations (Supplementary Figure S2). Values increased somewhat in subsurface SWIR sediments in the western part of the segment (Supplementary Figure S1B). In the top 5 cm of sediments the amount of chlorophyll-*a* (Chl-a) and its contribution to total CPE increased in the stations located inside the SWIR, with highest values at A3 (Table 1). In subsurface SWIR sediments the CPEs increased westward, whereas the Chl-a contribution to CPEs decreased (Supplementary Figure S1B).

Diffusive porewater flux rates are listed in Table 2. Fluxes of sulfide, DIC, ammonium and nitrate were observed at A3 and A3m, with the former showing the strongest fluxes. A weak upward flux of ammonium was also observed in the subsurface sediments at A2. Nitrate fluxes into the sediment were observed at the majority of sites inside the axial valley (A3m, A3, and A2), but not at the reference sites (N0, S0).

**Table 2:** Diffusive flux rates (J) of various geochemical constituents in the SWIR ridge valley areas A2 and A3.

Station	Sample ID	Environment	Constituent	Ds	J	Flux direction
				m^2^ a^-1^	mmol m^-2^ a^-1^	
PS81/659	A2	Surface (0–20 cm)	Nitrate	2.58 10^-2^	-14	Downward (upper 5.0 cm)
PS81/656	A2	Subsurface (50–550 cm)	Sulfate	1.33 10^-2^	-1	Downward
			Ammonium	2.58 10^-2^	1	Upward
PS81/661	A3	Surface (0–20 cm)	Sulfide	2.54 10^-2^	34	Upward
			DIC	1.38 10^-2^	600	Upward
			Ammonium	2.58 10^-2^	138	Upward
			Nitrate	2.58 10^-2^	-8	Downward (upper 5.5 cm)
PS81/657	A3	Subsurface (50–550 cm)	Sulfate	1.33 10^-2^	-4	Downward
			Sulfide	2.54 10^-2^	0.8	Upward
			DIC	1.38 10^-2^	5	Upward
			Ammonium	2.58 10^-2^	1	Upward
PS81/636	A3m	Surface (0–45 cm)	Sulfide	2.59 10^-2^	10	Upward
			DIC	1.37 10^-2^	32	Upward
			Ammonium	2.59 10^-2^	14	Upward
			Nitrate	2.58 10^-2^	-17	Downward (upper 3.5 cm)

*Ds, sediment diffusion coefficient; J, diffusive flux rate; DIC, dissolved inorganic carbon.*

### Microbial Abundances and Activity

Total cell numbers in the sediments, as determined by AODC (acridine orange direct counts), ranged between 0.25 cells × 10^9^ cells ml^-1^ wet sediment and 1.4 × 10^9^ cells ml^-1^ wet sediment (Table 3). Highest numbers were detected at the northern reference site (N0) and slightly lower in the axial valley. The lowest cell numbers were found at the station A3m. The CARD-FISH efficiency ranged from 43 to 67% (the sum of bacterial and archaeal cells counts relative to the total cell counts determined by AODC) and the number of active cells showed the same pattern as the AODC results. Bacteria with a median relative abundance of 48 ± 14% dominated over Archaea (3 ± 1%) at all stations (Table 3).

**Table 3:** Benthic microbial abundances and activity.

Station	Sample ID	Layer	Replicate	AODC	Bacteria	Archaea	DCF	
		cm		Cells × 10^9^ mL^-1^	%	%	nmol C mL^-1^ d^-1^	fmol C cell^-1^ d^-1^
PS81/681	N0	0–1	R1	1.2	36.8	3.8	3.25 (0.39; 9)	5.01 ( ×10(-3
			R2	1.4	54.3	3.1		
PS81/626	S0	0–1	R1	1.2	45.4	2.7	2.44 (0.06; 3)	3.75 × 10^-3^
			R2	1.2	58.6	1.9		
PS81/649	A1	0–1	R1	0.9	39.4	3.1	3.02 (1.18; 9)	6.96 × 10^-3^
			R2	0.9	51.0	2.5		
PS81/659	A2	0–1	R1	0.9	35.0	3.7	1.34 (0.14; 6)	3.25 × 10^-3^
			R2	0.8	55.5	3.3		
PS81/639	A2m	0–1	R1	0.6	65.1	1.1	0.64 (0.08; 3)	1.60 × 10^-3^
			R2	0.6	64.8	2.9		
PS81/636	A3m	0–1	R1	0.2	18.8	1.7	3.00 (0.42; 3)	28.34 × 10^-3^
			R2	0.2	35.9	0.8		

*mL, mL of wet sediment. Proportional bacterial and archaeal abundances are relative to the total cell counts (AODC). Cells counts from two sub-replicates of the core collected in each station are reported. The average and in parenthesis standard deviation and number of observations, respectively, are reported for dark carbon fixation (DCF) rates.*

Rates of dark carbon fixation (DCF) were highest at N0 and lowest at A2m (Table 3). However, we did not identify any clear pattern between stations outside and inside the ridge. Similar trends were observed for microbial activity per cell, with exception of A3m that showed a value up to 15 times higher than the other stations (0.03 fmol C cell^-1^ d^-1^).

### Microbial Alpha-Diversity

Rarefaction analysis showed that we captured more than 90% of bacterial and archaeal diversity of non-singletons at the stations investigated, as the rarefaction curves reached a plateau for Hill diversity indices H_1_ and H_2_, both in surface and subsurface samples (Supplementary Figure S3). Bacterial diversity indices did not show any apparent patterns between stations both in surface and subsurface layers (Supplementary Table S1A); H_1_ and H_2_ were higher in surface than in subsurface sediments (Supplementary Table S1A). Archaeal communities showed a substantially lower diversity than bacterial communities, which could be due to lower intragenomic heterogeneity of the amplified 16S rRNA gene region compared to region V3–V4 of Bacteria (Sun et al., 2013; Oton et al., 2016). Archaeal diversity indices in surface sediments were more than two times higher at A3 with the reduced sediments than at all the other stations, and H_1_ and H_2_ decreased westward in subsurface sediments (Supplementary Table S1B).

### Differences in Microbial Community Composition

**FIGURE 3:**
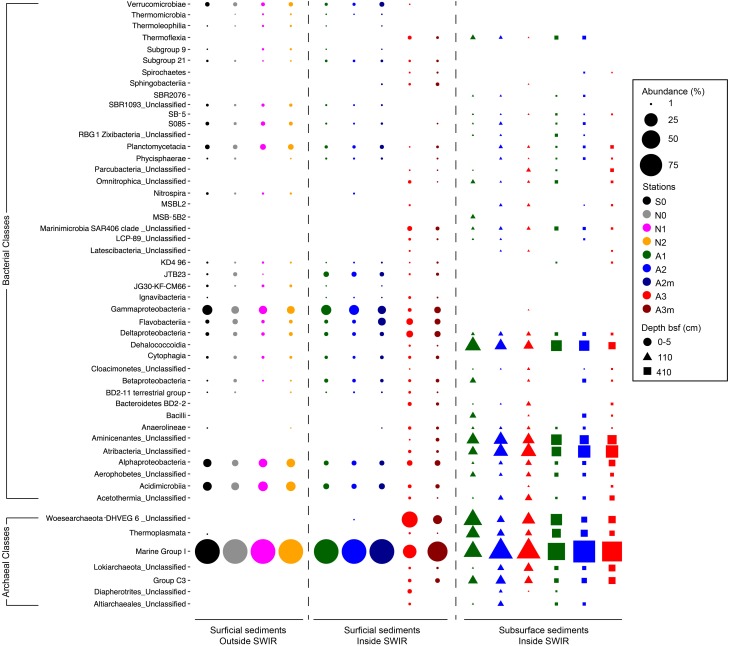
Bacterial and archaeal community composition in surface and subsurface sediments. Relative sequence abundance of **(A)** bacterial and **(B)** archaeal 16S rRNA at Class level resolution. Dominant community is here showed (i.e., community composed by those taxa present in the dataset with a relative abundance higher than 1%). For those taxa unclassified at Class level the Phylum is reported.

**FIGURE 4:**
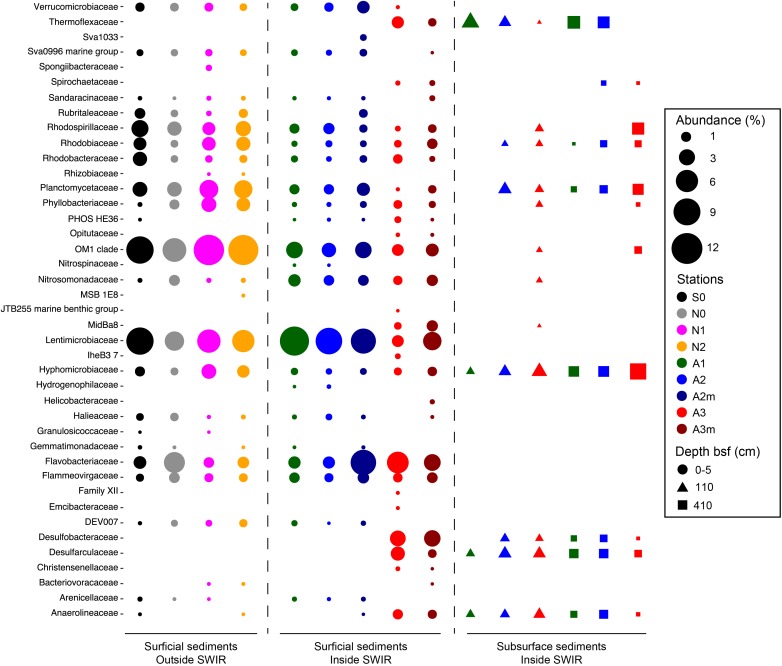
Bacterial community composition in surficial and subsurface sediments at Family level. Here we reported Families responsible for differences between bacterial communities inside and outside the SWIR and between surficial and subsurface bacterial communities. Families were selected after PCA analysis carried out on Hellinger transformed dominant bacterial and archaeal taxa (i.e., OTU > 0.1%) comparing (i) 0–5 cm layer of A3–A3m versus all stations, (ii) 0–5 cm layer of stations inside SWIR (A1 and A2–A2m) versus outside SWIR stations (N0, N1, N2, and S0), and (iii) 0–5 cm layer of all stations versus subsurface layer of SWIR stations.

The most important bacterial classes in the surface layer (0–5 cm) of the areas outside the rift valley (S0, N0, N1, and N2), which alone constituted 23–36% of the total sequence abundance in each sample, were Gammaproteobacteria, Acidimicrobiia and Alphaproteobacteria (Figure 3). Stations A1, A2, and A2m on the SWIR were dominated by these taxa as well (14–18%), but also by JTB23, Betaproteobacteria, Deltaproteobacteria, Flavobacteria, and Verrucomicrobiae, which all together represented between 31 and 36% of the total microbial community. The SWIR stations A3 and A3m exhibited a higher diversity of the most abundant taxa, i.e., about 37% of the bacterial community were represented by Atribacteria, Bacteroidetes BD2-2, “*Ca.* Marinimicrobia” (SAR406), Omnitrophica, Thermoflexia, Aminicenantes, Cytophagia, Sphingobacteriia, Acetothermia, Anaerolineae, BD2-11 terrestrial group, Ignavibacteria, JG30-KF-CM66, LCP-89 and Subgroup 21, in addition to the taxa already mentioned above. Dissimilarities in bacterial community composition between stations in area A3 and other stations were mainly explained by Bacteroidetes BD2.2, Thermoflexia, Dehalococcoidia, “*Ca.* Marinimicrobia” (SAR406), Anaerolineae, Atribacteria, Parcubacteria, Pla3 Lineage, Spirochaetes, and Deltaproteobacteria (Supplementary Figure S4). Specifically, surface sediments of Area 3 were enriched in Desulfobacteraceae (mostly SEEP-SRB1), Desulfarculaceae (mostly Desulfatiglans), Thermoflexaceae (Thermoflexus), Spirochaetaceae, and mostly at A3m, also by Helicobacteraceae (Sulfurimonas and Sulfurovum) (Figure 4 and Supplementary Table S2). In all subsurface samples the dominant bacterial taxa were Dehalococcoida, Atribacteria, and Aminicenantes (Figure 3). Archaea were dominated by Marine Group I in all surface samples (63–98% of the archaeal sequences), except for station A3, where the dominant taxon was Woesearchaeota (DHVEG-6) with a relative sequence proportion of 39% (Figure 3). Furthermore, in Area 3 and in subsurface sediments the archaeal communities were also composed of Diapherotrites, Altiarchaeales, Lokiarchaeata, Thermoplasmata, and Group C3.

**FIGURE 5:**
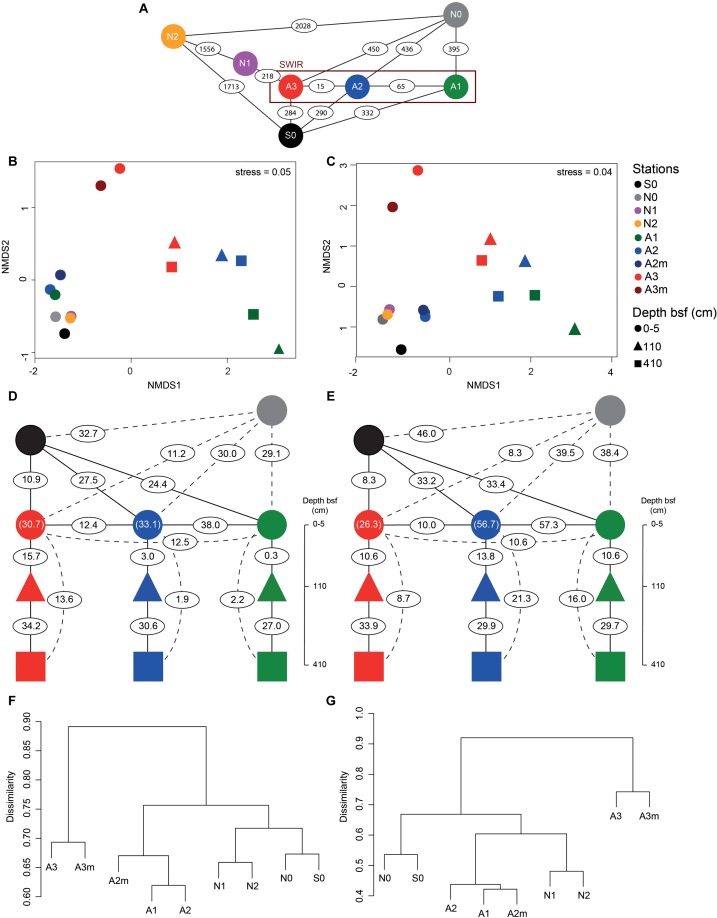
Beta-diversity of bacterial and archaeal communities in surface and subsurface sediments. **(A)** Geographic distance in kilometers between the investigated stations. Non-metric multidimensional scaling (nMDS) using Bray–Curtis distance on Hellinger transformed **(B)** bacterial and **(C)** archaeal community structure at OTU level. Percentage of **(D–F)** bacterial and **(E–G)** archaeal shared OTU between stations as defined by Jaccard dissimilarity matrix based on presence/absence OTU table (with 100 sequence re-samplings per sample on the smallest dataset); the numbers in parentheses represent the shared OTU between A2/A2m (green blue) and A3/A3m (red circle). For full matrices see Supplementary Table S3. bsf, below the seafloor.

Microbial community composition in surface sediments differed significantly between stations outside and inside the SWIR (ANOSIM, *r* > 0.40, *p* < 0.05); these differences were more pronounced when Area 3 (A3–A3m) was considered as a discrete group (ANOSIM, *r* > 0.90, *p* < 0.01; Figure 5B,C). At station A3 both bacterial and archaeal communities shared the lowest number of OTUs with all other stations (13 ± 2% and 12 ± 3%, respectively; Figure 5D-G and Supplementary Table S3). Bacterial and archaeal communities in surface sediments of SWIR stations (A1, A2, and A2m) shared a higher number of OTUs with each other (36 ± 2% and 57 ± 3%, respectively) than with communities outside the SWIR (27 ± 2% and 40 ± 5%, respectively; Figure 5D–G and Supplementary Table S3). Surface and subsurface bacterial and archaeal communities were significantly different (ANOSIM, *r* = 0.98 and *r* = 0.61, respectively, *p* = 0.001; Figure 5B,C), with the highest number of shared OTUs between 110 and 410 cm layers (Figure 5D,E and Supplementary Table S3). Shared bacterial OTUs between 110 cm layer and the top 0–5 cm layer increased remarkably westward (from 0.3 to 16%), whereas shared archaeal OTUs did not show any clear pattern between stations (11–14%). In surface sediments of Area 3, 46 ± 8, 89 ± 1, and 4 ± 1% of OTUs affiliated with the taxa Dehalococcoida, Atribacteria, and Woesearchaeota were shared with subsurface sediments; this corresponds to 46 ± 12, 93 ± 2, and 12 ± 4% of total sequences assigned to them in surficial sediments, respectively. In surface sediments the number of both bacterial and archaeal unique OTUs increased at the stations A3 and A3m compared to the stations outside the SWIR (Supplementary Table S1). About 3–8% of all bacterial OTUs were unique to one station, with highest values at A3m (Supplementary Table S1A). Unique archaeal OTUs represented 16–34% and 1–3% of total OTUs in surface sediments of Area 3 (A3 and A3m) and other stations, respectively (Supplementary Table S1B). In subsurface samples the contribution of bacterial and archaeal unique OTUs ranged between 3 and 6% and between 2 and 7%, respectively.

### Factors Controlling Microbial Community Structure

The variables chlorophyll *a* (Chl-a), total organic carbon (TOC), and dissolved inorganic carbon (DIC) did not show collinearity (VIF < 4) and were therefore used as descriptors of changes in the environmental setting at different stations. DIC, ammonia and sulfide were significantly positively correlated (Pearson *r* > 0.95, *p* < 0.001), thus we used DIC as a proxy for the presence of porewater flux and the availability of reduced compounds.

**FIGURE 6:**
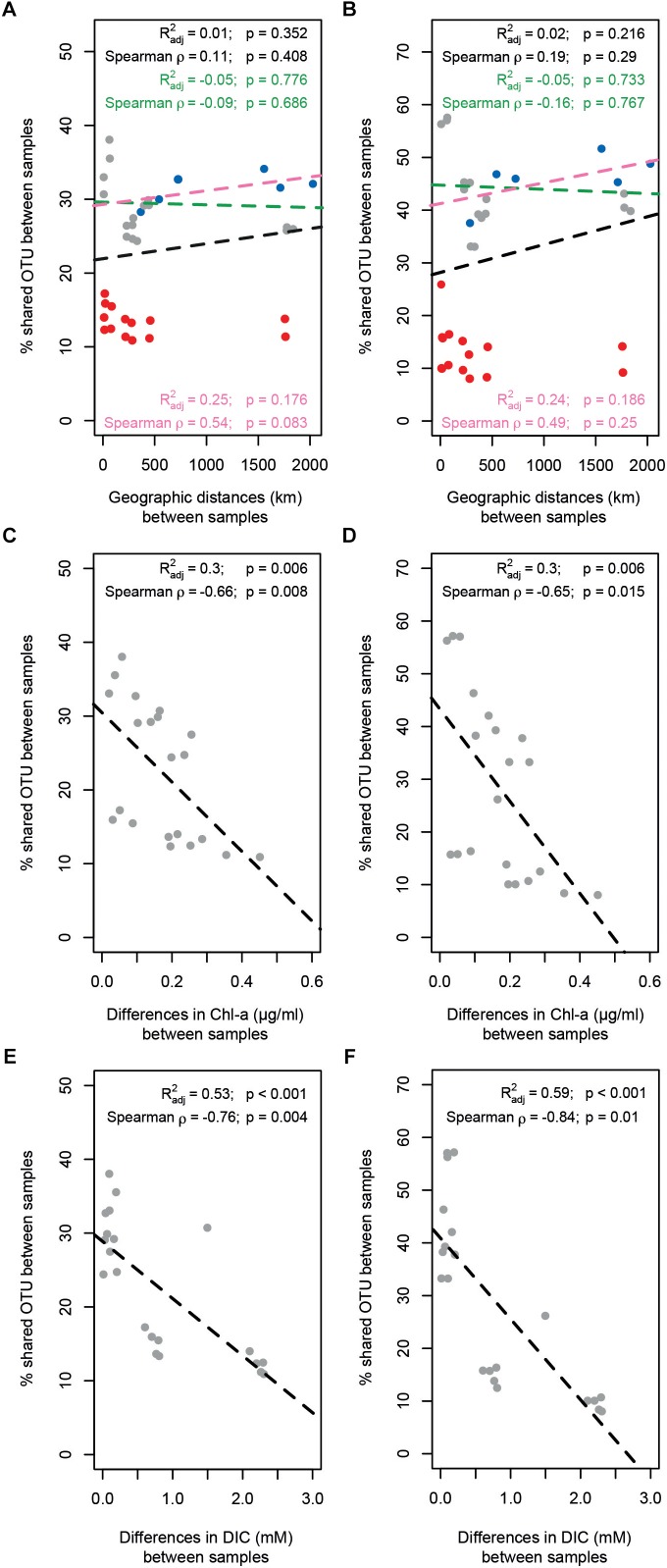
Relationship between geographic distance, environmental patterns and similarity in surface microbial community composition. The proportion of **(A)** bacterial and **(B)** archaeal shared OTUs between samples did not show any significant relationship with geographic distance; three different scenarios have been evaluated: full data-set including all stations (all dots; black text and dotted line); excluding stations in Area 3 (red dots; green text and dotted line); considering only the stations outside SWIR (blue dots; pink text and line). The proportion of **(C–E)** bacterial and **(D–F)** archaeal shared OTUs between samples decreased significantly with differences in chlorophyll *a* content (Chl-a; **C,D**) and dissolved inorganic carbon concentration (DIC; **E,F**). Dotted lines are linear model fits. Linear models’ *R*^2^, Spearman’s rho correlations, and their significance (Mantel tests with 1000 permutations) are reported in each panel.

Bacterial and archaeal community similarity (i.e., proportion of shared OTUs) between samples did not show significant relationships with geographic distance, even when stations at Area 3 were excluded or only stations outside the SWIR (N0, N1, N2, and S0) were considered (Figure 6A,B). In contrast, the proportion of bacterial and archaeal OTUs shared between stations (not including N1 and N2) were significantly related with differences in the environmental setting (Spearman ρ = 0.63 and *p* < 0.01, Spearman ρ = 0.66 and *p* < 0.05), and correlated negatively with differences in Chl-a content and DIC concentration (Supplementary Table S4 and Figure 6C–F). The extent of the relationship between Chl-a and OTU variations was different between stations inside and outside the ridge, whereas the variations related to DIC concentration were mostly related with larger differences between stations in Area 3 and other stations (similarity < 20%). Accordingly, a combination of Chl-a and DIC concentrations explained 46 and 61% of the variance in bacterial and archaeal community structure, respectively (Supplementary Table S4). Both bacterial and archaeal community structures were mainly explained by DIC (23 and 29%, respectively) rather than Chl-a (10 and 20%, respectively; Supplementary Figure S5).

## Discussion

Ocean ridges are the largest continuous topographic feature on Earth, representing diverse geobiological habitats including hydrothermal vents, different types of hard and soft bottom including typical pelagic sediments (Orcutt et al., 2011). Where active venting occurs, substantial energy may be delivered to specific chemosynthetic groups of bacteria that provide the basis of a food web independent of photosynthetically produced matter, including symbiotic interactions with animals. MOR rocks host their own microbial communities that add substantial diversity to this deep-sea realm (Santelli et al., 2008). However, the specific role of non-hydrothermal MOR sediments in shaping benthic microbial diversity and connectivity in the deep sea is largely unknown. Furthermore, the increasing interest in mining seafloor massive sulfide (SMS) deposits at oceanic ridges calls for a better baseline knowledge about the diversity, variability and connectivity of benthic biological communities, in order to assess and forecast significant ecological impacts (Boetius and Haeckel, 2018; Miller et al., 2018). In this study, the diversity of bacterial and archaeal 16S rRNA genes at the sedimented SWIR segment 10°–17°E and in the adjacent sedimentary seafloor north and south of the ridge (Figure 1) within a water depth range of 3,655–4,869 m were investigated to test whether the SWIR may act as a physical barrier (i) reducing the benthic north-south microbial genetic flux in the South Atlantic Polar Front and (ii) promoting isolation of microbial communities inside the ridge. The topography (high ridge flanks and deep valley) and axial orientation (parallel to the main eastward bottom water flow) of SWIR limit the northward and southward flow of bottom water. The SWIR segment studied here is located in a region with relatively high sedimentation rates for an open ocean system (ca. 1–30 cm kyr^-1^ in the last 200,000 years; Nürnberg et al., 1997; Mackensen et al., 2001). This, together with the V-shape topography at this site, produced a high sediment thickness (ca. 80 m) at the bottom of the axial ridge valley (Supplementary Figure S6). The area is within a relatively productive biogeochemical province (ca. 51.7 × 10^6^ km^2^) represented by an annual productivity of 8.4 mol C m^-2^ yr^-1^ and an estimated carbon flux of 85.8 mmol C m^-2^ yr^-1^ to the seafloor at 4,487 m depth (Seiter et al., 2004; Watling et al., 2013). The higher accumulation of Chl-a inside the SWIR compared to outside (up to 10 times; Table 1), and the increase of Chl-a toward the SWIR deepest axial stations (Supplementary Figure S1) is likely an effect produced by the V-shaped topographyat this site.

The detection of significant differences in bacterial and archaeal community structure at stations located outside and inside the SWIR supports at first sight the idea of the MOR acting as a physical barrier limiting microbial dispersal (Figure 5). Nevertheless, stations to the north of SWIR were not more similar to each other than with the station to the south (e.g., N1–N2 vs. N0–S0; Figure 5F,G). As described above, sedimentary matter composition and porewater chemistry indicated substantial differences in biogeochemistry, especially in the stations A3 and A3m. The environmental setting explained a large fraction of variance in bacterial and archaeal communities (45 and 61%, respectively; Supplementary Figure S5 and Supplementary Table S4), indicating environmental selection rather than isolation-by-distance as a main controlling factor.

The presence of the negative relationship between community similarity and differences in sedimentary chlorophyll *a* (Figure 6C,D) suggests food availability as a major driver of differences in bacterial and archaeal community composition between stations. This type of relationship has been shown before for deep-sea sediments in the Arctic Ocean (Bienhold et al., 2012; Jacob et al., 2013) and the primary role of trophic resource availability in shaping deep-sea benthic microbial community structure globally has been substantiated in more recent studies (Bienhold et al., 2016; Danovaro et al., 2016). This is reflected by the increase in the relative abundance of specific taxa related to phytoplankton/complex organic matter degradation at the SWIR stations in comparison to adjacent northern and southern sites (Figure 4). This includes the class Flavobacteriia, which has also shown positive correlations with chlorophyll pigments in an Arctic region (Bienhold et al., 2012). Flavobacteria have been associated with the ability to hydrolyze complex plant polymers (Humphry et al., 2001; Knoll et al., 2001; Williams et al., 2013). More specifically, the relative sequence abundance of the genus *Flavobacterium* was considerably higher at SWIR stations than in the adjacent seafloor. Pelagic and deep-sea benthic members of this taxon were previously shown to respond positively to phytoplankton blooms (Teeling et al., 2012, 2016; Ruff et al., 2014; Vigneron et al., 2017), indicating their potential role in complex phytodetritus matter degradation. Another class that seems to be selected for by higher organic matter availability at SWIR stations is Verrucomicrobiae; members of this class are known to play a role in polysaccharide degradation (Cardman et al., 2014; Li et al., 2016).

The different porewater biogeochemistry in Area 3 explained the majority of the observed differences in community composition between stations A3 and A3m with other stations (Figure 6E,F). Area 3 is located in the deepest axial section of the ridge valley where the large sink of organic matter can enhance early diagenesis processes (D’Hondt et al., 2002) and where the underlying lithosphere (i.e., peridotite) can promote serpentinization processes (Schmid and Schlindwein, 2016). These features produce an unusual geological setting in the deep sea, which is likely responsible for the observed upward efflux of anoxic porewater enriched in reduced compounds (Table 2), such as ammonia and sulfide in the surface sediments and potentially methane and hydrogen in deepest subsurface sediments. Hence, the highest per-cell microbial activity measured at A3m (Table 3) may be a consequence of the availability of multiple energy sources. During the PS81 expedition, no evidence for recent hydrothermal activity was found, neither at the seafloor nor in the water column of the amagmatic accretionary SWIR segment studied here (Schlindwein, 2014). Only one large veneroid bivalve of the family Vesicomyidae (genus *Christineconcha*, identified by Sergei Galkin, IORAS) was discovered, that had crawled onto an ocean bottom seismometer, which was deployed close to stations A3m 1 year before (Schlindwein, 2014; Supplementary Figure S6). Such Vesicomyids (i.e., genus *Christineconcha*) are known to inhabit reduced sediments with sulfide fluxes (Krylova and Von Cosel, 2011; Decker et al., 2012).

The most remarkable difference to other sites was the higher relative sequence proportion of potential sulfate-reducing bacteria (i.e., Desulfobacteraceae and Desulfarculaceae), which represented about 7% of all bacterial sequences at station A3 compared to less than 0.01% at stations outside the SWIR (Figure 3). This indicates that the high amount of organic carbon and the anaerobic conditions at Area 3 may support the development of sulfate-reducing communities, which are typically found in subsurface OM rich continental margin and cold-seep sediments (Teske, 2010). Interestingly about 35% of sequences related to potential sulfate reducers belonged to the genus SEEP-SRB1 (Supplementary Table S2), which is a sulfate-reducing partner of anaerobic methanotrophic archaea ANME-2 (Schreiber et al., 2010) and dominates methane-rich sediments (Ruff et al., 2015); however, we did not detect ANME-2 sequences in any of our samples. Most of the SEEP-SRB1 sequences found in Area 3 were closely related to sequences found in other chemosynthetic habitats and in SWIR vent fields (Supplementary Figure S7). Hydrogen sulfide, the catabolic product of sulfate reducers, probably favored sulfur-oxidizing bacteria in surface sediments of Area 3 (i.e., Helicobacteraceae), where their relative sequence proportion (ca. 0.2–1%; Figure 4) increased up to 500 times compared to outside the ridge. Sulfur oxidizers are a fundamental component of hydrothermal chemosynthetic communities and Helicobacteraceae dominate benthic chemolithotrophic communities at rocky hydrothermal vents of SWIR (Ding et al., 2017) and worldwide MOR (e.g., Flores et al., 2011; Sievert and Vetriani, 2012; Meier et al., 2017).

In surface sediments of Area 3, Dehalococcoida and Atribacteria were another important component of bacterial assemblages. These two taxa also dominated subsurface bacterial communities (Figure 3) and they are typically reported from subsurface environments associated with methane hydrates, hydrocarbon seeps, and petroleum reservoirs (Webster et al., 2004; Inagaki et al., 2006; Pham et al., 2009; Orcutt et al., 2011; Kobayashi et al., 2012; Parkes et al., 2014). There was no evidence for the presence of hydrocarbons in our sediments, and methane was only measured at nanomolar concentrations (Table 1), thus the presence of Dehalococcoida and Atribacteria in surface sediments could be due to an upward transport of porewater flux from deeper sediment layers. The high permeability and porosity of sediment in the ridge axial valley would allow for porewater circulation that could be responsible for an increased connectivity between subsurface and surface bacterial communities in this area compared to the more consolidated sediments outside the ridge (Figure 5D).

Area 3 also differed in archaeal diversity (Supplementary Figure S3 and Supplementary Table S1B) and community structure (Figure 3, 5C) from the other stations. The increased diversity and endemism of archaeal populations of Area 3 (Supplementary Table S1B) was associated with a decrease in their relative abundance, which was less than 2% of total microbial cells (Table 3). The dominance of Marine group I (Thaumarchaeota) suggests that archaea could have a primary role in nitrification (Könneke et al., 2005; Molari et al., 2013). However, the contribution of this taxon to total archaeal sequences did not increase in Area 3 where we found the highest concentrations of ammonia. Marine group I members use oxygen to oxidize ammonia, thus they may be limited by oxygen availability under the anoxic conditions in Area 3. Nevertheless, Marine group I was also a dominant group in the investigated anoxic subsurface layers, as reported for other deep-sea subsurface sediments (e.g., Durbin and Teske, 2010), which may mean that the metabolic diversity and ecological niche of this archaeal taxon is not fully elucidated yet (Offre et al., 2013). A large proportion of archaeal sequences found in surface sediments of Area 3 and subsurface layers of other SWIR stations belonged to Woesearchaeota (Figure 3), an enigmatic archaeal group of the monophyletic DPANN superphylum (Rinke et al., 2013; Castelle et al., 2015). Woesearchaeota have been detected in various environments including marine hydrothermal habitats (Takai and Horikoshi, 1999; Li et al., 2015), subsurface marine sediments, acid mines, groundwater (Baker et al., 2010; Castelle et al., 2015; Shcherbakova et al., 2016), high-altitude lakes (Ortiz-Alvarez and Casamayor, 2016), and recently in SWIR hydrothermal chimneys (Ding et al., 2017). Recent genome reconstructions support an anaerobic heterotrophic lifestyle (Liu et al., 2018), and their small genome size and the fact that most of the core biosynthetic pathways were partial or absent also suggest that Woesearchaeota have a host-associated/syntrophic or parasitic lifestyle, maybe even with bacteria (Baker et al., 2010; Castelle et al., 2015) or methanogenic Archaea (Liu et al., 2018). Despite the limited information about the ecological role of Woesearchaeota, the presence of this archaeal taxon in surface sediments of Area 3 supports connectivity between surface and anaerobic subsurface environments.

## Conclusion

Our study suggests that the amagmatic SWIR fragment investigated here substantially enhanced microbial diversity by providing additional biogeochemical niches, foremost via sediment accumulation in the axial valley. Accordingly, variations in microbial community composition were driven by changes in trophic resource availability and the biogeochemical setting at this site. In the axial valley, porewater circulation also promoted connectivity between subsurface and surface communities, and favored microbial taxa typically associated with reduced sediments, including such found at hydrothermal vent fields on the SWIR and in other deep-sea regions. This indicates a potential role of ultraslow spreading ridges in connecting spatially isolated chemosynthetic communities in the deep sea, instead of inducing isolation. In this regard the findings reported here expand our knowledge about microbial community dynamics in these systems, and stimulate future research to better elucidate the role of magma-starved ridges in deep-sea biodiversity and connectivity.

## Author Contributions

AB and MM designed the study and performed field observation and sampling activities. MM carried out cell counts and analyzed biogeochemical data. GV analyzed Illumina sequencing data and calculated the phylogenetic tree. FS calculated porewater fluxes. MM and GV made statistical analysis. MM, GV, CB, FS, and AB interpreted the results and wrote the manuscript.

## Conflict of Interest Statement

The authors declare that the research was conducted in the absence of any commercial or financial relationships that could be construed as a potential conflict of interest.

## Footnotes

1 https://doi.org/10.1594/PANGAEA.877640
