# Supplementary material for: Microbial Diversity and Connectivity in Deep-Sea Sediments of the South Atlantic Polar Front
Source: Front Microbiol. 2019 Apr 9;10:665. doi: 10.3389/fmicb.2019.00665 (PMC6465420; doi:10.3389/fmicb.2019.00665)
Supplement: Supplementary file 1 [file Data_Sheet_1.PDF]

Supplementary Material

Microbial diversity and connectivity in deep-sea sediments of the South Atlantic Polar Front

Gilda Varliero, Christina Bienhold, Florian Schmid, Antje Boetius and Massimiliano Molari*

*** Correspondence:** Massimiliano Molari: mamolari@mpi-bremen.de

List Supplementary Materials:

- **Figure S1. Biogeochemical profiles of SWIR subsurface sediments:** (**A**) the porewater profiles of dissolved components and (**B**) organic matter and chloroplastic pigment equivalents (CPEs) profiles. In the sulfide plot values for station A3 (PS81/661) refer to the bottom x-axis, for all other stations the values refer to the top x-axis. The % of chlorophyll *a* refers to the contribution of chlorophyll *a* to total CPEs
- **Figure S2. Organic matter and chloroplastic pigment equivalents (CPEs) profiles of surface sediments (0−5 cm) outside and inside the SWIR.** The grey (southern reference) and black lines (northern reference) represent outside SWIR stations, the colored lines inside samples. The % of chlorophyll *a* refers to the contribution of chlorophyll *a* to total CPEs.
- **Figure S3. Rarefaction curves and diversity coverage** for bacterial communities in surface (0−5 cm; **A**−**E**) and subsurface (110 cm and 410 cm; **B**−**F**) sediments and archaeal communities in surface (0−5 cm; **C**−**G**) and subsurface (110 cm and 410 cm; **D**−**H**) sediments.
- **Figure S4. Two-dimensional PCA with bacteria surface samples at Class level resolution**. Hellinger transformed dominant community (i.e. community composed by those OTUs that represent more than 0.1% of the total number of sequences in each sample) was used for the analysis. The darker the variance arrows, the more their associated values explain the variability shown in the graph. List of abbreviation: Aceto = Acetothermia; Aci = Acidimicrobiia; Alpha = Alphaproteobacteria; Amini = Aminicenantes; Anea = Anaerolineae; Atri = Atribacteria; Bact = Bacteroidetes; BD2.11 = BD2.11 terrestrial group; BD2.2 = Bacteroidetes BD2.2; Beta = Betaproteobacteria; Chla = Chlamydiae; Chlo = Chloroflexi; Cloaci = Cloacimonetes; Cyano = Cyanobacteria; Cyt = Cytofagia; Deha = Dehalococcoidia; Delta = Deltaproteobacteria; Epsi = Epsilonbacteria; Flavo = Flavobacteriia; Gamma = Gammapoteobacteria; Gemma = Gemmatimonadetes; Holo = Holophagae; Igna = Ingnavibacteria; JG30 = JG30.KF.CM66; Late = Latescibacteria; Mol = Mollicutes; Nitros = Nitrospira; Omni = Omnitrophica; Opi = Opitutae; Parcu = Parcubacteria; PAUC43f = PAUC43f marine benthic group; Phyci = Phycisphaerae; Pla = Planctomycetacia; Pla3 = Pla3 Lineage; SAR406 = Marinimicrobia SAR406 clade; Soli = Solibacteres; Sphi = Sphingobacteriia; Spiro = Spirochaetes; SR1 = SR1 Absconditabacteria; Sub21 = Subgroup.21; Sub9 = Subgroup 9; Thefle = Thermoflexia; Theph = Thermoleophilia; Ther = Thermomicrobia; TM6 Dependentiae = TM6; VC2.1.Bac22 = Bacteroidetes VC2.1.Bac22; Ver=Verrucomibrobiae. For unclassified groups we used the "_U" suffix.
- **Figure S5. Partitioning of the biological variation** in (**A**) bacterial and (**B**) archaeal community structure at the OTU level between chlorophyll *a* (Chl-a) and dissolved inorganic carbon (DIC). For ANOVA details see Table S3. * $p < 0.05$.
- **Figure S6. Vesicomyidae bivalve and RV Polarstern's sub-bottom profiler data. (A)** Zoom

in of Area 3 showing location of sediment-echosounder profiles (red and green lines) and bivalve in respect to sites A3 and A3m. (**B**) Picture sowing the living specimen of veneroid bivalve of the family Vesicomyidae and genus Christineconcha (identified by Sergei Galkin, IORAS) that was retrieved attached to an ocean bottom seismometer close to coring location PS81/636. Sediment-echosounder data showing (**C**) laminated strata up to 80 m thickness above the crystalline basement at A3m (station PS81/636), and (**D**) 40 m layered strata of pelagic sediments above deeper structures, that may either be of sedimentary or magmatic origin, at A3 (station PS81/661 and PS81/657).

- **Figure S7. Phylogenetic tree of SEEP-SRB1.** The tree backbone was calculated considering only the full-length reference 16S rRNA gene sequences (>900 bp). Maximum Likelihood Method based tree with 1000 bootstrap replicates was performed. Short 16S rDNA generated in this study by Illumina platform were added with Parsimony Method and highlighted in bold. For these sequences, relative abundance is displayed distinguishing by site and sediment layer. Branch points with bootstrap values > 80% are indicated with filled circles.
- **Table S1. Diversity indices and unique OTUs** for (**A**) bacterial and (**B**) archaeal communities. Indices and unique OTUs were calculated without singletons.
- **Table S2. Dominant bacterial Genera (cut-off > 0.5%) in surficial and subsurface sediments.**
- **Table S3. Percentage of shared OTUs** between bacterial (**A** and **B**) and archaeal (**C** and **D**) communities in surficial (**A** and **C**) and subsurface (**B** and **D**) sediments at investigated stations. Shared OTUs were calculated with 100 sequence re-samplings per sample on the smallest dataset, and average (white area) and standard deviation (grey area) are given.
- **Table S4. Output linear model (LM) and redundancy analysis (RDA) with their ANOVAs.**

1 Supplementary Figures and Tables

1.1 Supplementary Figures

Figure S1. Biogeochemical profiles of SWIR subsurface sediments: (**A**) the porewater profiles of dissolved components and (**B**) organic matter and chloroplastic pigment equivalents (CPEs) profiles. In the sulfide plot values for station A3 (PS81/661) refer to the bottom x-axis, for all other stations the values refer to the top x-axis. The % of chlorophyll *a* refers to the contribution of chlorophyll *a* to total CPEs.

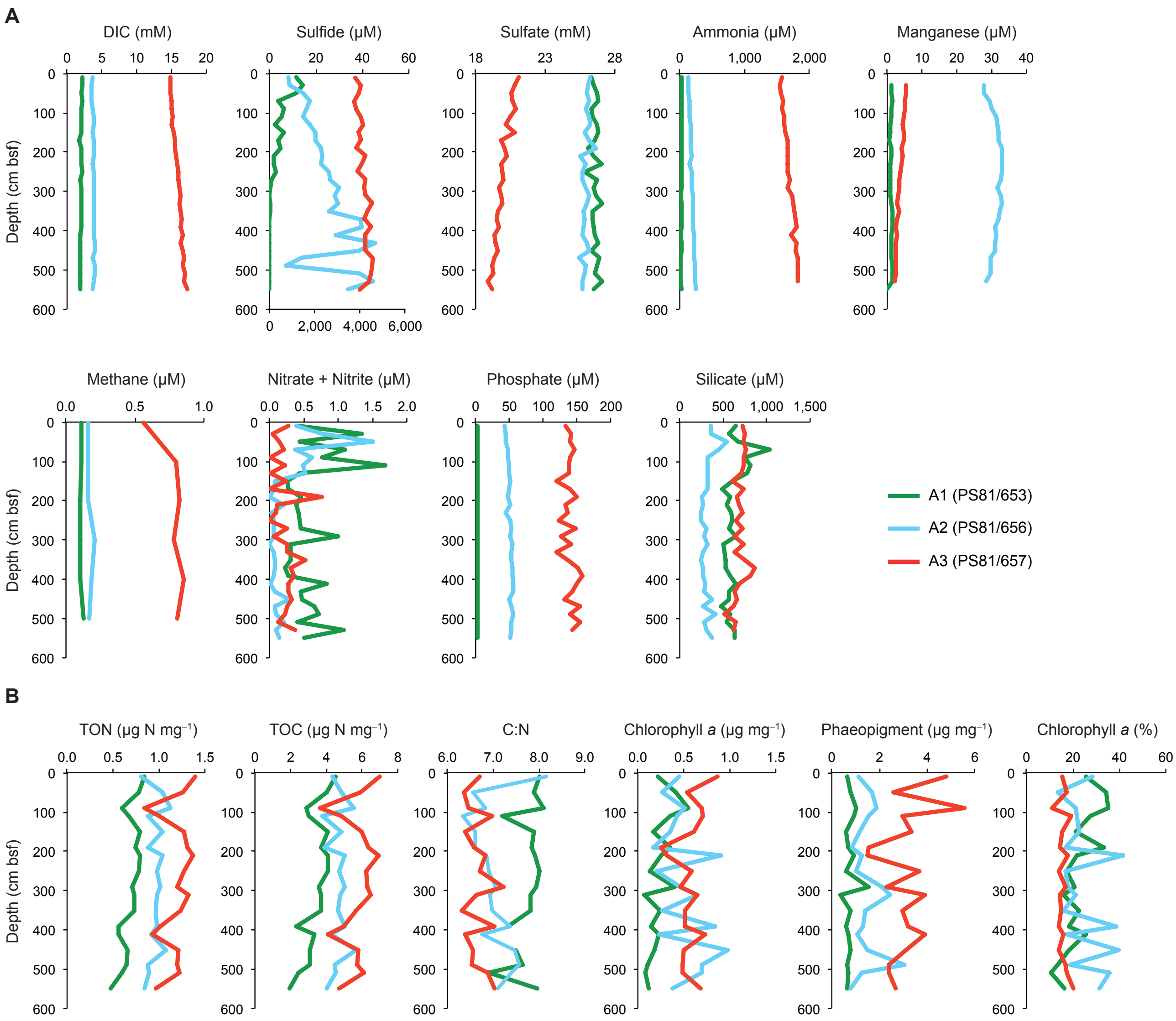

DIC: dissolved inorganic carbon; TON: total organic nitrogen; TOC: total organic carbon; C:N: total organic carbon and nitrogen ratio; cm bsf: centimeters below seafloor.

Figure S2. Organic matter and chloroplastic pigment equivalents (CPEs) profiles of surface sediments (0−5 cm) outside and inside the SWIR. The grey (southern reference) and black lines (northern reference) represent outside SWIR stations, the colored lines inside samples. The % of chlorophyll *a* refers to the contribution of chlorophyll *a* to total CPEs.

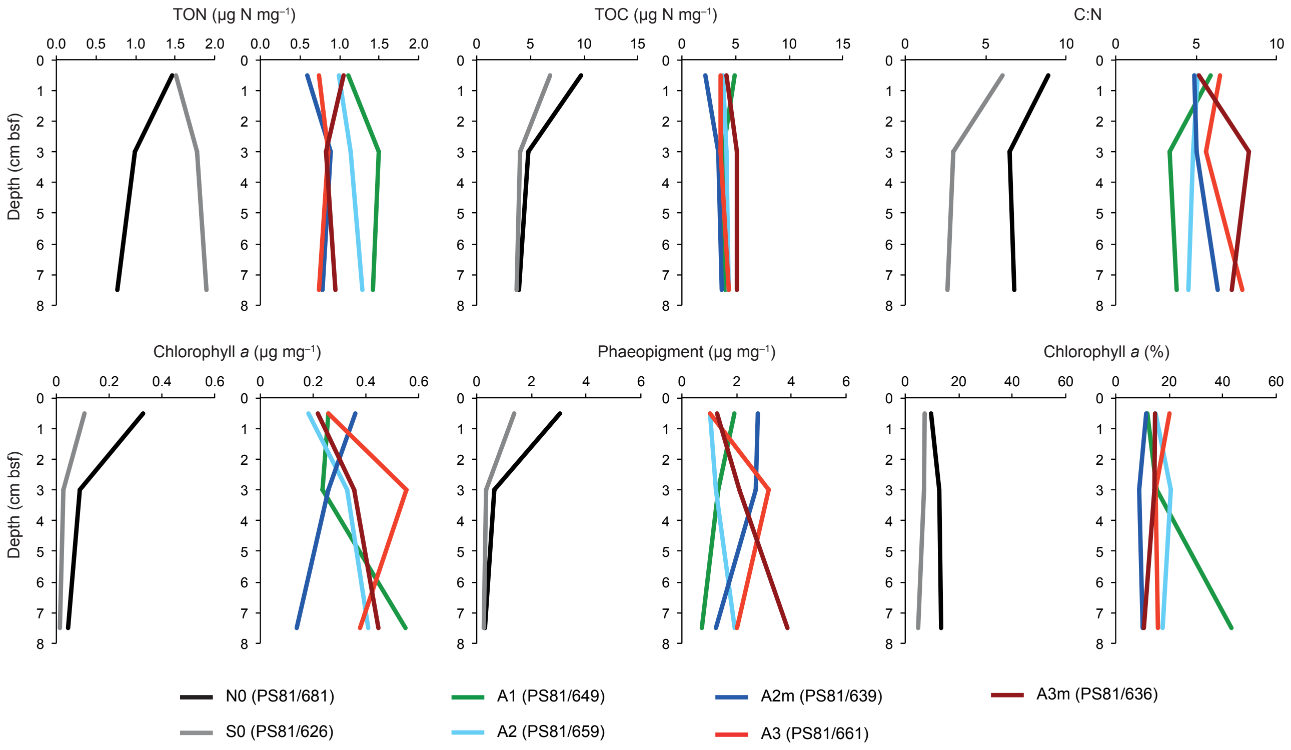

DIC: dissolved inorganic carbon; TON: total organic nitrogen; TOC: total organic carbon; C:N: total organic carbon and nitrogen ratio; cm bsf: centimeters below seafloor.

Figure S3. Rarefaction curves and diversity coverage for bacterial communities in surface (0−5 cm; **A−E**) and subsurface (110 cm and 410 cm; **B−F**) sediments and archaeal communities in surface (0−5 cm; **C−G**) and subsurface (110 cm and 410 cm; **D−H**) sediments.

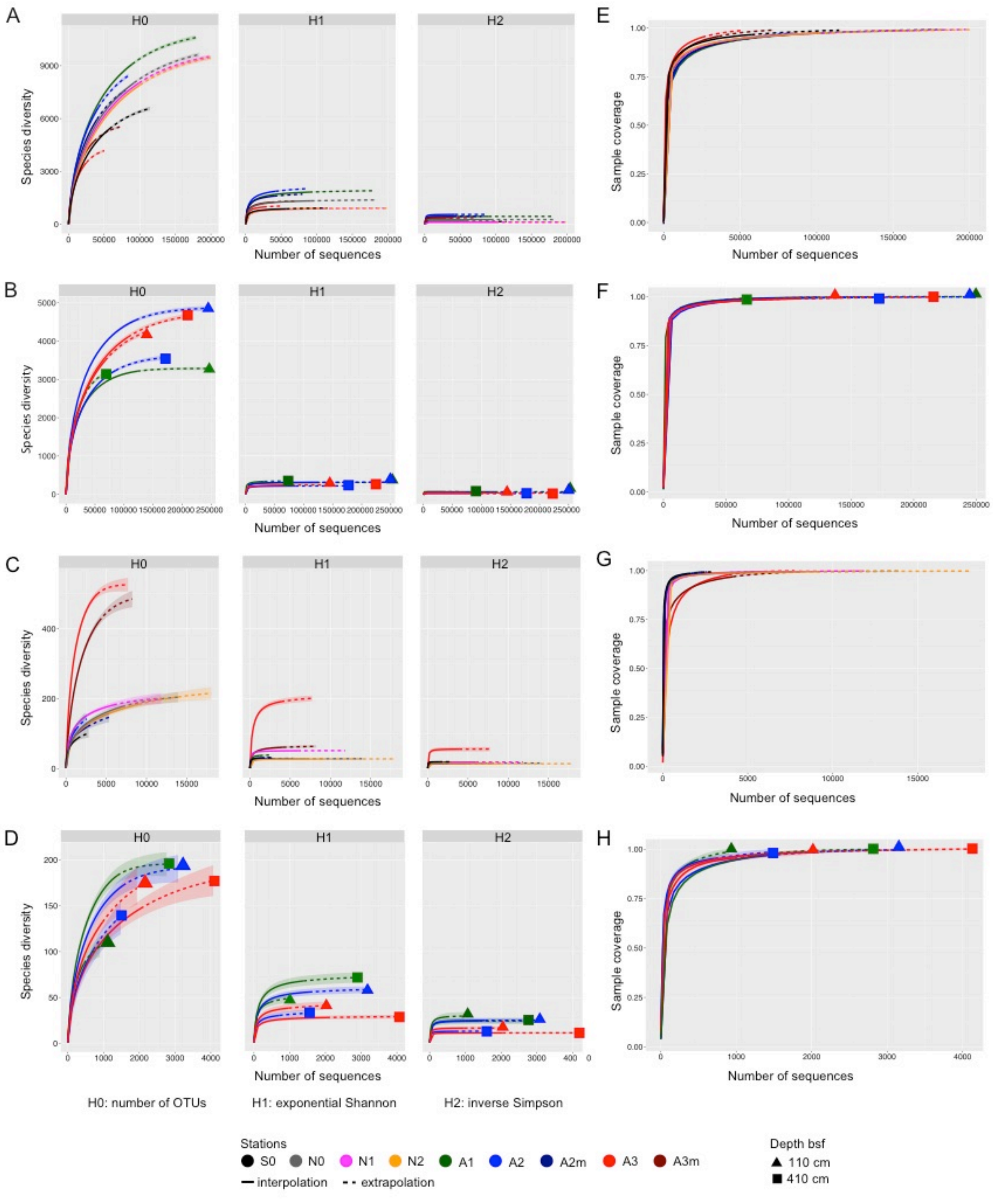

Figure S4. Two-dimensional PCA with bacterial surface samples at class level resolution. Hellinger transformed dominant community (i.e. community composed by those OTUs that represent more than 0.1% of the total number of sequences in each sample) was used for the analysis. The darker the variance arrows, the more their associated values explain the variability shown in the graph. List of abbreviation: Aceto = Acetothermia; Aci = Acidimicrobiia; Alpha = Alphaproteobacteria; Amini = Aminicenantes; Anea = Anaerolineae; Atri = Atribacteria; Bact = Bacteroidetes; BD2.11 = BD2.11 terrestrial group; BD2.2 = Bacteroidetes BD2.2; Beta = Betaproteobacteria; Chla = Chlamydiae; Chlo = Chloroflexi; Cloaci = Cloacimonetes; Cyano = Cyanobacteria; Cyt = Cytofagia; Deha = Dehalococcoidia; Delta = Deltaproteobacteria; Epsi = Epsilonbacteria; Flavo = Flavobacteriia; Gamma = Gammapoteobacteria; Gemma = Gemmatimonadetes; Holo = Holophagae; Igna = Ingnavibacteria; JG30 = JG30.KF.CM66; Late = Latescibacteria; Mol = Mollicutes; Nitros = Nitrospira; Omni = Omnitrophica; Opi = Opitutae; Parcu = Parcubacteria; PAUC43f = PAUC43f marine benthic group; Phyci = Phycisphaerae; Pla = Planctomycetacia; Pla3 = Pla3 Lineage; SAR406 = Marinimicrobia SAR406 clade; Soli = Solibacteres; Sphi = Sphingobacteriia; Spiro = Spirochaetes; SR1 = SR1 Absconditabacteria; Sub21 = Subgroup.21; Sub9 = Subgroup 9; Thefle = Thermoflexia; Theph = Thermoleophilia; Ther = Thermomicrobia; TM6 Dependentiae = TM6; VC2.1.Bac22 = Bacteroidetes VC2.1.Bac22; Ver=Verrucomibrobiae. For unclassified groups we used the "_U" suffix.

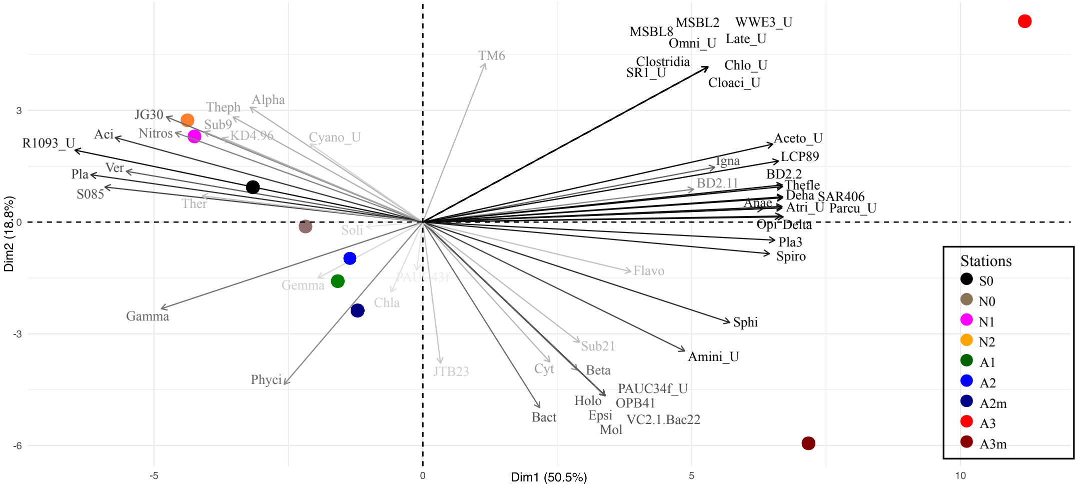

Figure S5. Partitioning of the biological variation in (**A**) bacterial and (**B**) archaeal community structure at the OTU level between chlorophyll *a* (Chl-a) and dissolved inorganic carbon (DIC). For ANOVA details see Table S3. * $p < 0.05$.

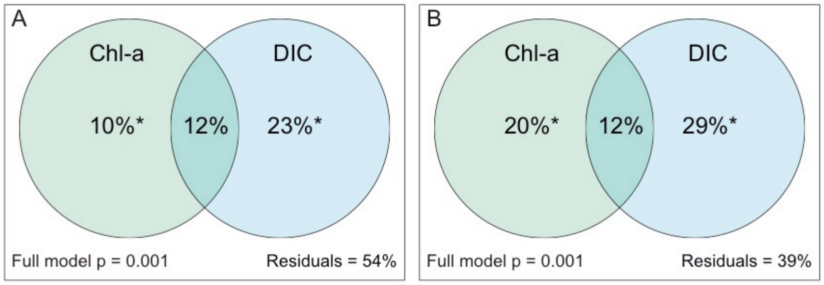

Figure S6. Vesicomyidae bivalve and RV Polarstern's sub-bottom echo sounder profiles (Atlas Parasound P-70). (A) Zoom in of Area 3 showing location of sediment-echosounder profiles (red and green lines) and bivalve in respect to sites A3 and A3m. (**B**) Picture sowing the living specimen of veneroid bivalve of the family Vesicomyidae and genus Christineconcha (identified by Sergei Galkin, IORAS) that was retrieved attached to an ocean bottom seismometer close to coring location PS81/636. Sediment-echosounder profiles showing (**C**) laminated strata up to 80 m thickness above the crystalline basement at A3m (station PS81/636), and (**D**) 40 m layered strata of pelagic sediments above deeper structures, that may either be of sedimentary or magmatic origin, at A3 (station PS81/661 and PS81/657).

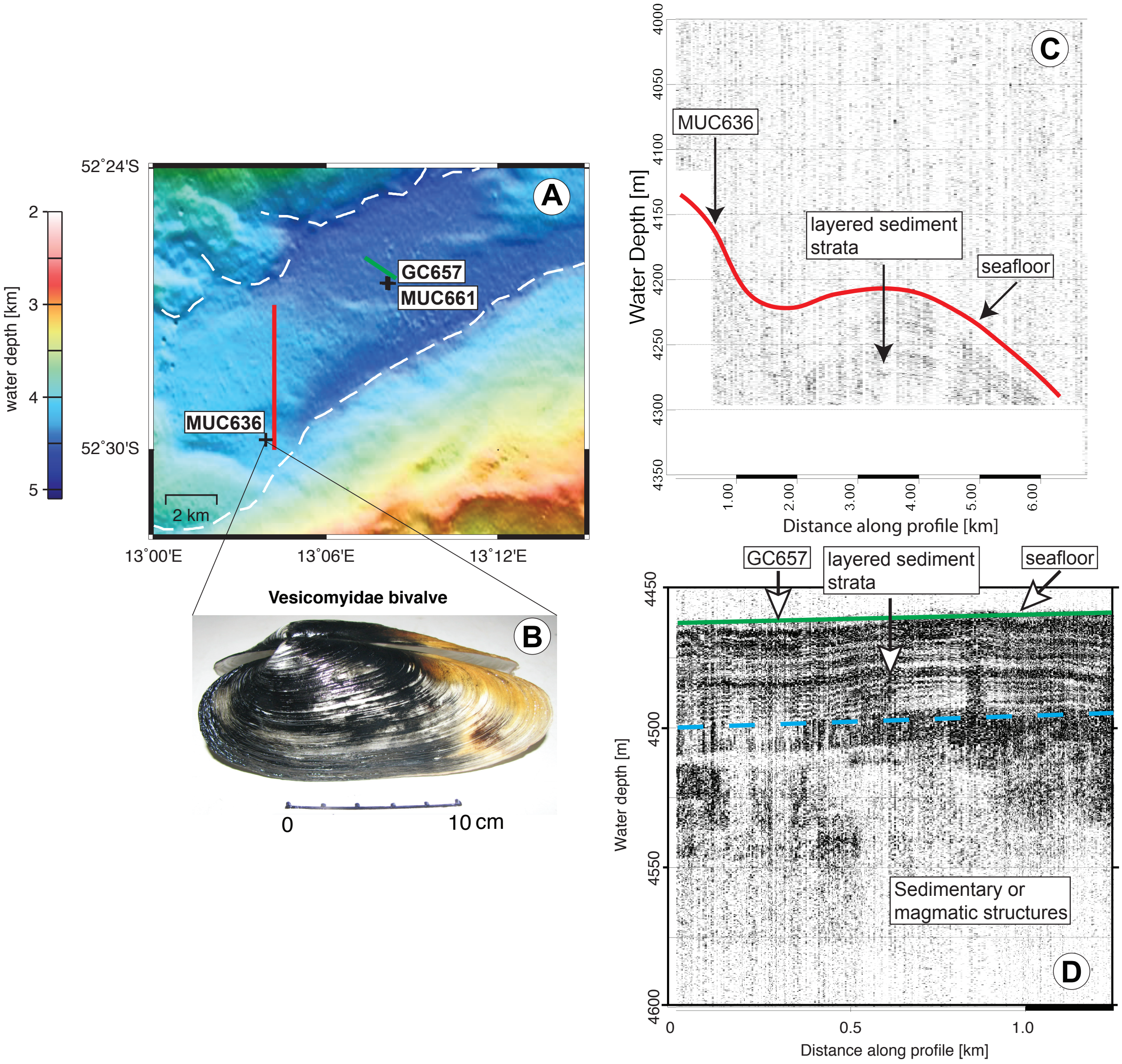

Figure S7. Phylogenetic tree of SEEP-SRB1. The tree backbone was calculated considering only the full-length reference 16S rRNA gene sequences (>900 bp). Maximum Likelihood Method based tree with 1000 bootstrap replicates was performed. Short 16S rDNA generated in this study by Illumina platform were added with Parsimony Method and highlighted in bold. For these sequences, relative abundance is displayed distinguishing by site and sediment layer. Branch points with bootstrap values > 80% are indicated with filled circles.

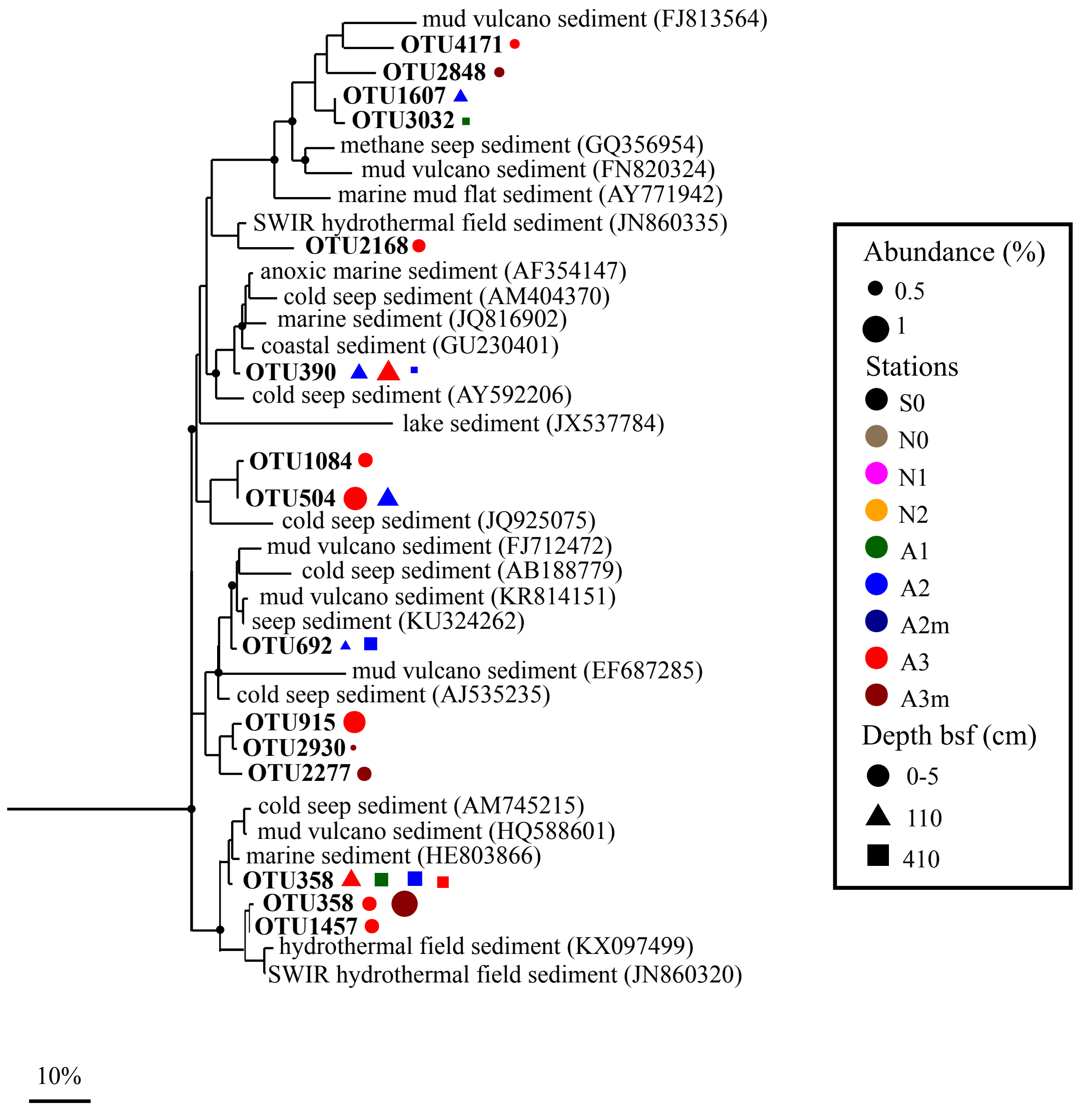

1.2 Supplementary Tables

Table S1. Diversity indices and unique OTUs for (**A**) bacterial and (**B**) archaeal communities. Indices and unique OTUs were calculated without singletons.

A

	Sequences n. [a]	H_0	H_1	H_2	Chao1 [b]	sd	Unique (%) [b]	sd
N1	139440	8019	884	116	6504	138	5	0.2
N2	139912	7912	881	120	6328	160	5	0.2
N0	139578	8032	1326	263	6536	136	5	0.1
S0	74704	5413	875	154	4821	92	4	0.1
A1	110793	9095	1822	442	7553	148	6	0.2
A2	54052	6639	1871	555	6116	104	5	0.1
A2m	50912	5957	1477	444	5530	99	4	0.1
A3	36019	3506	974	305	3407	45	6	0.1
A3m	49780	4718	1246	369	4451	61	8	0.1
A1_110	153104	3214	306	68	2792	64	6	0.1
A1_410	43342	2597	332	69	2483	42	3	0.1
A2_110	144469	4550	307	49	3759	106	5	0.1
A2_410	103271	3237	213	27	2798	81	3	0.1
A3_110	84143	3498	236	43	3032	76	3	0.1
A3_410	132729	4108	224	35	3351	95	4	0.1

B

	Sequences n. [c]	H_0	H_1	H_2	Chao1 [b]	sd	Unique (%) [b]	sd
N1	17410	182	39	18	152	15	3	0.6
N2	23159	184	27	14	132	18	2	0.5
N0	18423	177	28	15	136	22	2	0.5
S0	2280	86	30	19	83	7	2	0.4
A1	1996	114	37	18	111	9	1	0.4
A2	2133	113	30	13	109	8	1	0.3
A2m	4302	120	28	14	105	10	1	0.3
A3	7806	499	191	56	434	24	34	1.2
A3m	7692	422	61	14	363	36	16	1.1
A1_110	1391	87	45	29	84	7	5	0.6
A1_410	3017	183	68	24	159	19	7	0.8
A2_110	3278	172	56	25	146	20	7	0.8
A2_410	1415	102	30	13	95	13	2	0.4
A3_110	2143	132	38	17	117	19	4	0.7
A3_410	3176	147	28	11	116	21	4	0.7

H_0: number of OTUs; H_1: exponential Shannon; H_2: inverse Simpson; [a] after the merging of forward and reverse reads; [b] calculated with 100 sequence re-samplings per sample on the smaller dataset (surface / subsurface), average data and standard deviation (sd) are given; [c] after quality trimming of merged forward and reverse reads.

Table S2. Dominant bacterial Genera (cut-off > 0.5%) in surficial and subsurface sediments.

Genus	N1	N2	N0	S0	A1	A2	A2m	A3	A3m	A1_110	A2_110	A3_110	A1_410	A2_410	A3_410
Acetothermia_unclassified	0.0	0.0	0.0	0.0	0.0	0.0	0.0	1.4	0.3	0.3	2.5	4.8	0.8	1.5	3.8
Acidimicrobiales_unclassified	0.6	0.6	0.4	0.4	0.3	0.3	0.4	0.1	0.1	0.0	0.0	0.0	0.0	0.0	0.1
Acidobacteria_unclassified	2.3	2.3	1.3	1.7	1.8	1.8	1.5	1.1	1.1	0.0	0.0	0.0	0.0	0.0	0.0
Aerophobetes_unclassified	0.0	0.0	0.0	0.0	0.0	0.0	0.0	0.1	0.0	3.5	1.7	2.7	2.8	2.2	2.3
Aminicenantes_unclassified	0.0	0.0	0.0	0.0	0.0	0.0	0.0	0.4	1.9	15.9	19.0	14.2	18.7	13.0	13.9
Anaerolineaceae_unclassified	0.2	0.3	0.2	0.3	0.2	0.2	0.3	1.5	1.9	0.7	1.1	1.2	1.0	1.2	0.4
Aquibacter	0.8	1.1	2.7	0.8	1.3	0.9	2.7	2.7	1.8	0.0	0.0	0.0	0.0	0.0	0.0
Atribacteria_unclassified	0.0	0.0	0.0	0.0	0.0	0.0	0.0	1.7	1.2	8.7	19.8	22.9	14.9	24.3	25.4
Bacteria_unclassified	2.2	2.8	1.6	2.0	2.0	2.0	1.5	6.3	4.5	2.4	2.0	1.9	3.3	1.3	2.5
Bacteroidetes BD2-2_unclassified	0.0	0.0	0.0	0.0	0.0	0.0	0.0	3.0	2.0	0.1	0.2	2.1	0.2	0.1	0.9
Bacteroidetes VC2.1 Bac22_unclassified	0.0	0.0	0.0	0.0	0.0	0.0	0.0	0.2	1.3	0.0	0.0	0.0	0.0	0.0	0.0
BD7-8 marine group_unclassified	0.7	0.5	0.6	1.2	1.6	3.1	0.6	0.2	0.4	0.0	0.0	0.1	0.0	0.0	0.0
Blastopirellula	0.9	0.9	1.0	0.9	0.6	0.6	1.0	0.3	0.5	0.0	0.0	0.1	0.0	0.0	0.1
Candidatus Latescibacter	0.0	0.0	0.0	0.0	0.0	0.0	0.0	0.7	0.2	0.4	0.9	1.1	0.6	0.6	0.9
CCM11a_unclassified	0.3	0.3	1.0	0.7	1.1	1.3	1.2	0.3	1.2	0.3	0.6	0.2	0.7	0.9	0.1
Chloroflexi_unclassified	0.0	0.0	0.0	0.0	0.0	0.0	0.0	0.2	0.2	1.2	0.8	0.2	1.0	1.0	0.1
Cloacimonetes_unclassified	0.0	0.0	0.0	0.0	0.0	0.0	0.0	0.2	0.0	0.2	0.2	0.7	0.1	0.1	0.4
Cryomorphaceae_unclassified	0.1	0.1	0.6	0.3	0.6	0.7	0.7	0.1	1.3	0.0	0.0	0.0	0.0	0.0	0.0
Dehalococcoidia_unclassified	0.0	0.0	0.0	0.0	0.0	0.0	0.0	0.8	0.5	4.8	6.3	4.8	6.0	7.2	4.0
Deltaproteobacteria_unclassified	3.5	4.6	6.5	4.9	8.3	7.4	6.0	1.7	1.9	0.0	0.1	0.6	0.1	0.0	0.2
Desulfatiglans	0.0	0.0	0.0	0.0	0.0	0.0	0.0	3.7	1.6	0.9	1.8	1.5	1.7	1.7	1.0
Desulfobacteraceae_unclassified	0.0	0.0	0.0	0.0	0.0	0.0	0.0	1.2	1.3	0.0	0.0	0.1	0.0	0.1	0.1
Desulfobulbaceae_unclassified	0.0	0.0	0.0	0.0	0.0	0.0	0.0	0.0	0.0	0.6	0.0	0.0	0.1	0.0	0.0
DEV007_unclassified	1.9	2.0	2.2	1.1	1.7	1.4	1.5	0.3	0.3	0.0	0.0	0.1	0.0	0.0	0.1
Effusibacillus	0.0	0.0	0.0	0.0	0.0	0.0	0.0	0.0	0.0	3.8	0.0	0.5	0.0	2.3	0.7
Flammeovirgaceae_unclassified	1.4	1.4	2.4	1.5	2.1	2.0	2.4	1.4	1.9	0.0	0.0	0.0	0.0	0.0	0.0
Flavobacteriaceae_unclassified	1.5	1.6	4.5	2.2	2.1	2.0	6.1	5.2	2.9	0.0	0.0	0.1	0.0	0.0	0.0
FW22_unclassified	0.0	0.0	0.0	0.0	0.0	0.0	0.0	0.0	0.0	0.7	0.9	0.4	0.9	1.0	0.4
Gammaproteobacteria_unclassified	1.0	0.8	0.6	0.8	0.8	1.2	0.8	0.6	1.2	0.0	0.2	0.4	0.1	0.2	0.5
Gemmatimonadaceae_unclassified	0.3	0.4	0.6	0.5	0.5	0.5	0.5	0.2	0.4	0.0	0.0	0.0	0.0	0.0	0.0
Gemmatimonadetes_unclassified	1.0	1.2	1.4	1.4	1.8	2.1	1.4	1.5	1.4	0.1	0.2	0.1	0.2	0.1	0.0
GIF3_unclassified	0.0	0.0	0.0	0.0	0.0	0.0	0.0	0.6	0.2	1.9	2.8	0.9	2.8	3.0	1.0
GIF9_unclassified	0.0	0.0	0.0	0.0	0.0	0.0	0.0	0.1	0.0	0.3	0.4	0.9	0.9	0.4	1.0
Gracilibacteria_unclassified	0.0	0.0	0.0	0.0	0.0	0.0	0.1	0.7	0.2	0.0	0.0	0.1	0.0	0.0	0.1
Halieaceae_unclassified	0.1	0.1	0.4	0.2	0.4	0.6	0.2	0.1	0.2	0.0	0.0	0.0	0.0	0.0	0.0
Halioglobus	0.3	0.4	0.8	0.8	0.5	0.8	0.5	0.1	0.1	0.0	0.0	0.0	0.0	0.0	0.0
Hydrogenedentes_unclassified	0.1	0.2	0.3	0.3	0.5	0.5	0.5	0.4	0.4	0.0	0.0	0.2	0.0	0.0	0.1
Hyphomicrobiaceae_unclassified	2.9	2.0	0.8	1.3	0.7	0.5	0.6	0.9	1.2	0.5	1.1	1.9	1.4	1.5	3.5
Ilumatobacter	1.1	1.2	1.0	1.1	0.9	0.8	1.1	0.2	0.2	0.0	0.0	0.1	0.1	0.0	0.1
JG30-KF-CM66_unclassified	1.7	1.7	1.0	1.4	0.4	0.4	0.5	0.1	0.1	0.2	0.1	0.0	0.1	0.0	0.0
JTB23_unclassified	0.4	0.4	2.4	0.7	4.4	3.8	2.8	0.7	1.2	0.0	0.0	0.1	0.0	0.0	0.0
JTB255 marine benthic group_unclassified	9.6	9.1	7.9	12.3	15.4	13.7	11.3	3.5	6.7	0.0	0.0	0.1	0.1	0.0	0.0
KD4-96_unclassified	0.8	0.6	0.5	0.5	0.4	0.4	0.5	0.5	0.6	0.0	0.2	0.2	0.4	0.1	0.6
KI89A clade_unclassified	0.3	0.3	0.5	0.8	0.5	0.6	0.2	0.1	0.1	0.0	0.0	0.0	0.0	0.0	0.0
Latescibacteria_unclassified	0.2	0.3	0.3	0.3	0.4	0.4	0.4	0.9	0.5	0.1	0.1	0.1	0.1	0.1	0.1
LCP-89_unclassified	0.0	0.0	0.0	0.0	0.0	0.0	0.0	1.0	0.4	0.4	0.5	0.2	0.3	0.5	0.1
Lutibacter	0.0	0.0	0.0	0.0	0.2	0.1	0.2	0.3	0.9	0.0	0.0	0.0	0.0	0.0	0.0
Marinimicrobia (SAR406 clade)_unclassified	0.0	0.0	0.0	0.0	0.1	0.0	0.0	3.8	2.2	1.3	1.5	1.5	2.4	1.3	0.8
Mesorhizobium	2.7	2.2	1.1	0.0	0.0	0.0	0.0	0.0	0.0	0.0	0.0	0.0	0.0	0.0	0.0
MSB-5B2_unclassified	0.0	0.0	0.0	0.0	0.0	0.0	0.0	0.0	0.0	2.0	0.0	0.0	0.0	0.0	0.0
MSBL2_unclassified	0.0	0.0	0.0	0.0	0.0	0.0	0.0	0.5	0.0	0.0	0.8	1.0	0.0	0.1	0.6
MSBL5_unclassified	0.0	0.0	0.0	0.0	0.0	0.0	0.0	0.3	0.3	4.5	3.2	1.0	3.6	3.0	0.4
MSBL9_unclassified	0.0	0.0	0.0	0.0	0.0	0.0	0.0	0.5	0.3	0.5	0.9	0.3	0.5	0.3	0.3
Napoli-4B-65_unclassified	0.0	0.0	0.0	0.0	0.0	0.0	0.0	0.1	0.0	2.7	1.0	0.9	2.7	1.8	0.8
Nitrosomonas	0.4	0.4	1.6	0.3	2.0	1.5	1.5	1.2	1.8	0.0	0.1	0.4	0.0	0.0	0.2
Nitrospina	0.1	0.1	0.3	0.2	0.4	0.5	0.2	0.1	0.1	0.0	0.0	0.0	0.0	0.0	0.0
Nitrospira	0.9	0.8	0.8	0.8	0.4	0.7	0.1	0.0	0.1	0.0	0.0	0.0	0.0	0.0	0.0
ODP1230B30.02 sediment group_unclassified	0.0	0.0	0.0	0.0	0.0	0.0	0.0	0.6	0.3	0.1	0.3	0.4	0.0	0.1	0.5
OM1 clade_unclassified	13.2	12.9	8.2	10.6	4.1	3.4	3.9	2.5	2.7	0.0	0.1	0.6	0.2	0.1	1.0
OM182 clade_unclassified	0.1	0.1	0.2	0.2	0.2	0.5	0.1	0.0	0.0	0.0	0.0	0.0	0.0	0.0	0.0
OM190_unclassified	0.8	0.8	1.2	0.7	1.1	1.3	1.2	0.1	0.3	0.0	0.0	0.0	0.0	0.0	0.0
Omnitrophica_unclassified	0.0	0.0	0.1	0.1	0.1	0.1	0.1	4.3	2.4	5.7	2.5	2.4	5.1	2.0	2.0
OPB41_unclassified	0.0	0.0	0.0	0.0	0.0	0.0	0.0	0.2	0.3	0.1	0.5	0.7	0.2	0.4	0.8
Parcubacteria_unclassified	0.2	0.2	0.3	0.2	0.4	0.3	0.6	2.0	1.4	1.0	0.8	2.6	1.4	0.5	2.9
Pelagibius	0.4	0.6	0.4	0.7	0.2	0.3	0.1	0.1	0.1	0.0	0.0	0.0	0.0	0.0	0.0
Persicirhabdus	0.8	0.6	0.7	0.4	0.5	0.6	1.3	0.1	0.2	0.0	0.0	0.0	0.0	0.0	0.0
Phycisphaeraceae_unclassified	0.1	0.1	0.5	0.1	0.4	0.5	0.5	0.2	0.1	0.0	0.0	0.0	0.0	0.0	0.0
Phycisphaerales_unclassified	0.0	0.0	0.0	0.0	0.0	0.0	0.0	0.6	0.2	0.1	0.2	0.4	0.0	0.1	0.5
Pir4 lineage	5.5	5.5	3.5	3.9	2.1	2.1	2.9	1.2	2.3	0.2	2.0	1.4	0.9	1.0	2.3
Pla3 lineage_unclassified	0.1	0.1	0.3	0.1	0.3	0.3	0.4	1.3	1.7	0.1	0.6	0.5	0.1	0.3	0.4
Planctomyces	2.1	2.1	1.5	1.4	1.2	1.2	1.4	0.7	1.4	0.0	0.3	0.2	0.2	0.1	0.4
Planctomycetaceae_unclassified	2.9	2.5	2.1	1.9	1.5	1.5	1.9	0.9	1.8	0.1	0.8	0.7	0.7	0.7	1.4
Pseudahrensia	0.3	0.4	0.2	0.2	0.4	0.3	0.7	1.1	0.6	0.0	0.0	0.5	0.0	0.0	0.3
Pseudohongiella	0.1	0.0	0.2	0.1	0.3	0.6	0.1	0.0	0.2	0.0	0.0	0.0	0.0	0.0	0.0
Pseudomonas	0.0	0.0	0.0	0.9	0.0	0.0	0.0	0.0	0.0	0.0	0.0	0.0	0.0	0.0	0.0
Ralstonia	0.0	0.0	0.0	0.0	0.0	0.0	0.0	0.0	0.0	3.4	0.0	0.3	0.0	2.0	0.4
Rhizobiales_unclassified	0.4	0.4	0.2	0.2	0.4	0.3	0.4	0.6	0.4	0.0	0.1	0.5	0.1	0.1	0.6
Rhodobacteraceae_unclassified	0.8	1.2	1.0	2.8	0.6	0.7	0.6	1.7	0.7	0.0	0.0	0.4	0.0	0.0	0.4
Rhodobiaceae_unclassified	2.5	2.6	0.9	2.1	0.5	0.7	0.7	0.9	1.4	0.2	0.5	0.6	0.3	0.7	0.8
Rhodopirellula	0.5	0.5	0.3	0.5	0.3	0.5	0.3	0.1	0.2	0.0	0.1	0.1	0.0	0.1	0.2
Rhodospirillaceae_unclassified	3.2	3.8	4.3	5.2	3.2	3.8	2.2	0.9	1.6	0.0	0.3	0.9	0.1	0.3	2.4
Rhodothermaceae_unclassified	0.4	0.3	0.4	0.4	0.4	0.5	0.6	0.3	0.4	0.0	0.0	0.0	0.0	0.0	0.0
Roseibacillus	0.7	0.7	0.8	0.9	0.6	0.8	1.2	0.2	0.2	0.0	0.0	0.1	0.0	0.0	0.0
Rubritalea	0.7	1.2	1.1	1.5	0.2	0.3	1.1	0.1	0.1	0.0	0.0	0.1	0.0	0.0	0.0
S085_unclassified	2.8	2.2	1.3	1.9	0.5	0.5	0.7	0.2	0.2	0.8	1.4	0.0	0.7	1.1	0.1
Saprospiraceae_unclassified	0.2	0.3	0.4	0.3	0.5	0.5	1.0	0.1	0.1	0.0	0.0	0.0	0.0	0.0	0.0
SAR202 clade_unclassified	0.5	0.5	0.5	1.0	0.3	0.7	0.1	0.0	0.1	0.4	0.1	0.0	0.2	0.1	0.0
SAR324 clade(Marine group B)_unclassified	0.7	0.8	0.6	0.5	0.4	0.5	0.4	0.2	0.3	0.0	0.0	0.0	0.0	0.0	0.0
SB-5_unclassified	0.0	0.0	0.0	0.0	0.0	0.0	0.0	0.0	0.1	0.3	0.3	0.2	0.6	0.3	0.3
Sedimentitalea	0.3	0.3	0.3	0.6	0.3	0.2	0.5	0.5	0.3	0.0	0.0	0.2	0.0	0.0	0.1
SEEP-SRB1	0.0	0.0	0.0	0.0	0.0	0.0	0.0	3.2	3.4	0.5	1.1	0.6	0.9	0.9	0.3
Sh765B-AG-111_unclassified	0.0	0.0	0.0	0.0	0.0	0.0	0.0	0.1	0.0	10.1	2.9	2.2	5.2	4.0	2.2
Sphingobacteriales_unclassified	0.1	0.2	0.2	0.1	0.3	0.3	0.5	1.4	2.4	0.1	0.1	0.2	0.1	0.1	0.1
Spirochaeta 2	0.0	0.0	0.0	0.0	0.0	0.0	0.0	1.1	1.4	0.1	0.2	0.0	0.2	0.6	0.2
Subgroup 22_unclassified	0.8	0.7	0.7	0.7	1.6	1.5	1.0	0.2	0.3	0.0	0.0	0.1	0.0	0.0	0.1
Subgroup 26_unclassified	0.4	0.5	0.4	0.3	0.3	0.4	0.4	0.2	0.3	0.0	0.0	0.1	0.0	0.0	0.1
Sulfurimonas	0.0	0.0	0.0	0.0	0.0	0.0	0.0	0.0	0.7	0.0	0.0	0.0	0.0	0.0	0.0
Sva0725_unclassified	0.6	0.5	0.2	0.3	0.3	0.2	0.2	0.0	0.0	0.0	0.0	0.0	0.0	0.0	0.0
Sva0996 marine group_unclassified	1.2	0.9	0.9	0.8	1.1	1.0	1.3	0.5	0.3	0.0	0.0	0.1	0.0	0.0	0.1
Sva1033_unclassified	0.0	0.0	0.0	0.0	0.0	0.1	0.7	0.1	0.2	0.0	0.0	0.0	0.0	0.0	0.0
Thermoflexus	0.0	0.0	0.0	0.0	0.0	0.0	0.0	2.2	1.1	2.7	1.2	0.1	2.3	1.8	0.0
Ulvibacter	0.5	0.5	0.7	0.5	0.6	0.4	1.1	0.2	0.1	0.0	0.0	0.0	0.0	0.0	0.0
Unknown Family_unclassified	2.4	2.3	3.0	2.7	3.7	3.4	2.6	0.7	1.8	0.0	0.0	0.1	0.0	0.0	0.0
Urania-1B-19 marine sediment group	0.7	0.9	1.3	1.1	0.8	0.9	0.9	0.1	0.2	0.0	0.0	0.0	0.0	0.0	0.0
vadinBA26_unclassified	0.0	0.0	0.0	0.0	0.0	0.0	0.0	0.6	0.3	11.3	7.6	4.8	8.6	7.7	4.9
vadinHA49_unclassified	0.0	0.0	0.1	0.1	0.1	0.1	0.0	0.7	0.5	0.2	0.2	0.3	0.2	0.2	0.2
Verrucomicrobia_unclassified	0.2	0.3	0.3	0.1	0.2	0.3	0.3	0.4	0.6	0.1	0.1	0.1	0.0	0.0	0.1
Xanthomonadales_unclassified	0.7	0.5	0.5	0.6	0.7	0.6	0.3	0.1	0.2	0.0	0.0	0.0	0.0	0.0	0.0

Table S3. Percentage of shared OTUs between bacterial (**A** and **B**) and archaeal (**C** and **D**) communities in surficial (**A** and **C**) and subsurface (**B** and **D**) sediments at investigated stations. Shared OTUs were calculated with 100 sequence re-samplings per sample on the smallest dataset, and average (white area) and standard deviation (grey area) are given.

A

	N2	N1	N0	S0	A1	A2	A2m	A3	A3m
N2	–	0.5	0.4	0.4	0.4	0.4	0.3	0.2	0.2
N1	34.2	–	0.4	0.4	0.4	0.4	0.4	0.2	0.2
N0	32.1	30.0	–	0.4	0.4	0.4	0.4	0.2	0.2
S0	31.6	28.4	32.7	–	0.3	0.4	0.4	0.2	0.2
A1	26.0	26.5	29.1	24.4	–	0.4	0.4	0.2	0.3
A2	26.2	26.5	30.0	27.5	38.0	–	0.4	0.2	0.2
A2m	25.7	25.0	29.2	24.6	35.6	33.1	–	0.3	0.3
A3	11.4	11.3	11.2	10.9	12.5	12.4	14.0	–	0.4
A3m	13.8	13.8	13.6	13.3	15.5	15.9	17.2	30.7	–

B

	A1	A2	A3	A1_110	A2_110	A3_110	A1_410	A2_410	A3_410
A1	–	0.4	0.2	0.0	0.1	0.2	0.1	0.1	0.2
A2	38.1	–	0.2	0.0	0.1	0.2	0.1	0.1	0.2
A3	12.5	12.4	–	0.1	0.2	0.3	0.1	0.2	0.3
A1_110	0.3	0.3	2.0	–	0.3	0.2	0.5	0.3	0.2
A2_110	2.9	3.0	7.5	13.4	–	0.4	0.4	0.5	0.4
A3_110	6.1	5.9	15.7	6.6	18.5	–	0.3	0.4	0.6
A1_410	2.2	2.2	5.2	27.1	23.3	13.3	–	0.4	0.4
A2_410	1.9	1.9	5.8	15.5	30.6	17.1	24.7	–	0.4
A3_410	6.1	6.1	13.6	7.1	19.3	34.2	14.3	18.4	–

C

	N2	N1	N0	S0	A1	A2	A2m	A3	A3m
N2	–	3.3	3.2	2.8	2.8	3.1	3.0	0.7	1.2
N1	51.5	–	2.8	1.9	2.4	2.5	2.8	0.6	1.2
N0	48.4	46.7	–	3.0	2.0	3.0	2.6	0.6	1.1
S0	44.8	38.5	46.0	–	1.5	2.2	2.8	0.6	1.1
A1	39.9	45.3	38.4	33.4	–	2.8	3.0	0.7	1.2
A2	41.0	44.1	39.5	33.2	57.3	–	3.2	0.8	1.1
A2m	42.8	45.2	42.3	37.5	57.9	56.7	–	0.7	1.1
A3	9.2	9.6	8.3	8.1	10.6	10.0	10.0	–	1.5
A3m	14.1	15.1	13.7	12.4	16.3	15.6	15.7	26.3	–

D

	A1	A2	A3	A1_110	A2_110	A3_110	A1_410	A2_410	A3_410
A1	–	4.1	1.0	1.2	1.3	1.8	1.3	2.0	2.1
A2	49.9	–	1.1	1.2	1.4	2.1	1.7	2.1	1.9
A3	10.8	10.0	–	0.8	1.0	1.0	1.1	1.1	1.0
A1_110	10.6	10.5	5.2	–	1.6	1.3	2.7	2.0	1.5
A2_110	13.9	13.8	8.4	16.0	–	2.1	2.5	2.5	2.0
A3_110	18.3	16.8	10.6	11.6	21.6	–	1.7	2.5	3.1
A1_410	16.0	15.1	8.7	29.7	25.7	17.7	–	2.2	1.8
A2_410	21.3	21.3	9.1	19.8	29.9	24.5	25.0	–	2.7
A3_410	18.3	18.2	8.7	13.6	20.6	33.9	18.3	26.1	–

Table S4. Output linear model (LM) and redundancy analysis (RDA) with their ANOVAs.

		Bacteria				Archaea			
LM: PA.db ~ Env.db	adjR2	0.349				0.383			
	ANOVA	Df	SS	F	P	Df	SS	F	P
	**Env.db**	1	645.16	11.712	**0.003**	1	0.24945	13.45	**0.002**
	Residuals	19	1046.62			19	0.35238		
LM: PA.db ~ Chl-a.db+TOC.db+DIC.db	adjR2	0.505				0.564			
	ANOVA	Df	SS	F	P	Df	SS	F	P
	**Chl-a.db**	1	561.06	13.3937	**0.002**	1	0.202552	15.4434	**0.001**
	TOC.db	1	17.7	0.4225	0.524	1	0.003408	0.2599	0.617
	**DIC.db**	1	400.9	9.5704	**0.007**	1	0.172897	13.1823	**0.002**
	Residuals	17	712.12			17	0.222969		
RDA: ComStruc. ~ Chl-a+TOC+DIC	adjR2	0.387				0.647			
	ANOVA	Df	Variance	F	P	Df	Variance	F	P
	**Chl-a**	1	0.147063	3.49	**0.008**	1	0.138579	7.3923	**0.001**
	TOC	1	0.053125	1.2607	0.318	1	0.028552	1.5231	0.217
	**DIC**	1	0.085738	2.0347	**0.045**	1	0.095213	5.079	**0.008**
	Residual	3	0.126415			3	0.056239		
RDA: ComStruc. ~ Chl-a+DIC	adjR2	0.455				0.615			
	ANOVA	Df	Variance	F	P	Df	Variance	F	P
	Model	2	0.26261	3.5079	**0.001**	2	0.236748	5.7859	**0.001**
	Residual	4	0.14973			4	0.081836		
RDA: ComStruc. ~ Chl-a+Condition(DIC)	ANOVA	Df	Variance	F	P	Df	Variance	F	P
	Model	1	0.073066	1.952	**0.042**	1	0.074535	3.6431	**0.025**
	Residual	4	0.149728			4	0.081836		
RDA: ComStruc. ~ DIC+Condition(Chl-a)	ANOVA	Df	Variance	F	P	Df	Variance	F	P
	Model	1	0.11555	3.0869	**0.022**	1	0.098168	4.7983	**0.018**
	Residual	4	0.14973			4	0.081836		

PA.db: Jaccard dissimilarity matrix based on presence/absence OTU table (re-sampling); Env.db: Euclidean distance matrix based on standardized environmental variables; ComStruc.: Hellinger transformed community structure at OTU level; Chl-a: chlorophyll *a*; TOC: total organic carbon; DIC: dissolved organic carbon; adjR2: adjusted R^2; Df: degrees of freedom; SS: sum of the squares; F: statistic *F*; P: probability level.
